# Detecting and Characterizing Particulate Organic Nitrates with an Aerodyne Long-ToF Aerosol Mass Spectrometer

**DOI:** 10.1021/acsearthspacechem.2c00314

**Published:** 2022-12-22

**Authors:** Frans Graeffe, Liine Heikkinen, Olga Garmash, Mikko Äijälä, James Allan, Anaïs Feron, Manuela Cirtog, Jean-Eudes Petit, Nicolas Bonnaire, Andrew Lambe, Olivier Favez, Alexandre Albinet, Leah R. Williams, Mikael Ehn

**Affiliations:** † Institute for Atmospheric and Earth System Research/Physics, Faculty of Science, University of Helsinki, Helsinki00014, Finland; ‡ Department of Environmental Science and Bolin Centre for Climate Research, Stockholm University, StockholmSE-10691, Sweden; § Aerosol Physics Laboratory, Physics Unit, Tampere University, Tampere33014, Finland; ∥ Department of Earth and Environmental Sciences and National Centre for Atmospheric Science (NCAS), University of Manchester, Oxford Road, ManchesterM13 9PL, U.K.; ⊥ Univ Paris Est Créteil and Université Paris Cité, CNRS, LISA, Créteil, ParisF-94010, France; # Laboratoire des Sciences du Climat et de l’Environnement (LSCE), Gif-sur-Yvette91191, France; ∇ Aerodyne Research Inc., Billerica, Massachusetts01821, United States; ○ Institut National de l’Environnement Industriel et des Risques (INERIS), Verneuil-en-Halatte60550, France

**Keywords:** organo-nitrates, AMS, nitrate radicals, quantification, uncertainty, SOA

## Abstract

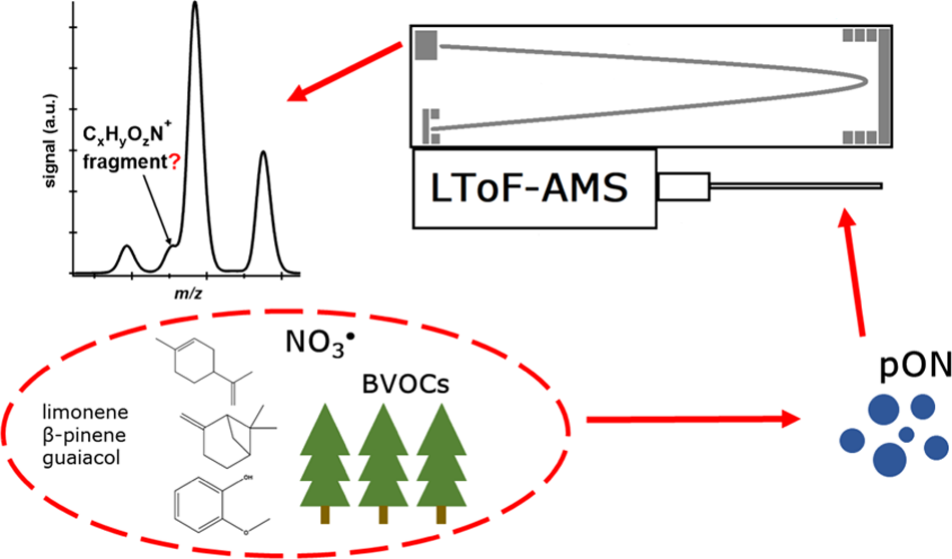

Particulate organic nitrate (pON) can be a major part of secondary organic aerosol (SOA) and is commonly quantified by indirect means from aerosol mass spectrometer (AMS) data. However, pON quantification remains challenging. Here, we set out to quantify and characterize pON in the boreal forest, through direct field observations at Station for Measuring Ecosystem Atmosphere Relationships (SMEAR) II in Hyytiälä, Finland, and targeted single-precursor laboratory studies. We utilized a long time-of-flight AMS (LToF-AMS) for aerosol chemical characterization, with a particular focus to identify C*_x_*H*_y_*O*_z_*N^+^ (“CHON^+^”) fragments. We estimate that during springtime at SMEAR II, pON (including both the organic and nitrate part) accounts for ∼10% of the particle mass concentration (calculated by the NO^+^/NO_2_^+^ method) and originates mainly from the NO_3_ radical oxidation of biogenic volatile organic compounds. The majority of the background nitrate aerosol measured is organic. The CHON^+^ fragment analysis was largely unsuccessful at SMEAR II, mainly due to low concentrations of the few detected fragments. However, our findings may be useful at other sites as we identified 80 unique CHON^+^ fragments from the laboratory measurements of SOA formed from NO_3_ radical oxidation of three pON precursors (β-pinene, limonene, and guaiacol). Finally, we noted a significant effect on ion identification during the LToF-AMS high-resolution data processing, resulting in too many ions being fit, depending on whether tungsten ions (W^+^) were used in the peak width determination. Although this phenomenon may be instrument-specific, we encourage all (LTOF-) AMS users to investigate this effect on their instrument to reduce the possibility of incorrect identifications.

## 1 Introduction

Secondary organic aerosol (SOA) constitutes a major fraction of atmospheric particulate matter (PM) around the globe,^1−3^ originating from the oxidation of volatile organic compounds (VOCs). Although the majority of VOCs are biogenic (BVOCs) in origin, the formation of SOA is dependent on the local sources of both biogenic and anthropogenic emissions. Nitrogen oxides, NO*_x_* = NO + NO_2_, are primarily emitted by anthropogenic sources,^4^ and they impact the atmospheric oxidant budget through participating in ozone formation (photochemical reactions involving VOC and NO*_x_*^5^) and nitrate (NO_3_) radical formation (from reactions of NO_2_ and O_3_^6,7^). A more direct link between NO*_x_* and SOA comes via reactions between VOCs and NO_3_ radicals. VOC + NO_3_ radical reactions can produce gas-phase organic nitrates (gON) that may have sufficiently low vapor pressure to condense onto particles and form particulate organic nitrate (pON).^8^ gON can also form via the minor pathway when organic peroxy radicals (RO_2_), for example, from VOC oxidation, react with NO.^9^ Several field measurements throughout the world, both in regions dominated by BVOCs and anthropogenic VOCs (AVOCs), have recognized pON as a substantial part of the submicron organic aerosol.^10−19^ Previous laboratory studies of different VOC + NO_3_ radical systems have regularly reported high SOA mass yields (several tens of percent, Table 2 in Ng et al.^8^), emphasizing the importance of these reactions.

The chemical composition of submicron aerosol is commonly measured in near-real-time by different versions of the Aerodyne Aerosol Mass Spectrometer (AMS) or the Aerosol Chemical Speciation Monitor (ACSM). Importantly, these aerosol mass spectrometers use electron impact ionization (70 eV), which is a hard ionization technique that causes substantial fragmentation of the sampled molecules. While this facilitates the bulk quantification of different aerosol species, neither AMS nor ACSM is capable of directly measuring the composition or concentration of pON. The AMS typically fragments the pON molecules into separate organic fragments, containing only C and H atoms (denoted here as CH^+^) or C, H, and O atoms (denoted as CHO^+^) and nitrate fragments (mainly the ions NO^+^ and NO_2_^+^).^20,21^ Fortunately, the ratio of the NO^+^ and NO_2_^+^ fragments in the AMS mass spectra differs between pON and inorganic ammonium nitrate (AN).^21^ This dependence in the NO^+^/NO_2_^+^ ratio can be utilized to quantify the fraction of aerosol nitrate present as pON. This method relies on known values of the NO^+^/NO_2_^+^ ratio for both AN and pON. While AN is routinely measured during standard AMS calibration, the NO^+^/NO_2_^+^ for pON is more difficult to determine as it depends on the pON precursors (see Section 3.2 for details). Although pON standards are becoming more readily available, they may not reflect the NO^+^/NO_2_^+^ ratios of the ambient PM of interest. Laboratory studies are needed to determine typical NO^+^/NO_2_^+^ ratios for different pON precursors. Despite these difficulties, the NO^+^/NO_2_^+^ ratio is a simple and robust method for estimating pON from ambient PM.^22^

Although the AMS cannot directly measure pON molecular formulae, small amounts of organic fragments that still retain N atoms (denoted here as CHON^+^) can be detected with the AMS if the mass resolving power of the instrument is sufficiently high.^23^ These CHON^+^ signals can be used as additional pON markers. High-resolution AMS data is analyzed with a custom peak fitting routine to fit overlapping ion peaks at a given *m*/*z*, based on a user-defined list of ions, *m*/*z* calibration, peak shape (PS), and peak width (PW).^23^ Importantly, the CHON^+^ fragment identification and quantification is highly sensitive toward data analysis uncertainties and errors.^24^ As the CHON^+^ fragment quantities are often small compared to other fragments at the same unit mass, the *m*/*z* calibration and PW must be precise for their accurate separation from other overlapping ions. The accuracy of both the *m*/*z* calibration and the PW determination is easily checked during the data analysis. Despite this, the PW might be under- or overestimated, depending on the selected ions.

In this study, we assess atmospheric concentrations and the diurnal behavior of pON measured by a long time-of-flight AMS (LToF-AMS), with a focus on evaluating the ability to observe CHON^+^ ions directly. The measurement site, the Station for Measuring Ecosystem Atmosphere Relationships (SMEAR) II, is situated in the boreal forest of Southern Finland, and can be considered ideal for investigating pON formation from BVOC oxidation under low AN loadings.^14,25,26^ The mass spectra collected from the field are further compared against the mass spectra of β-pinene, limonene, and guaiacol SOA that were generated during NO_3_ radical oxidation experiments in the laboratory. In addition, using the high mass resolution of the LToF-AMS, we investigate the effect of using tungsten ions (W^+^ ions) for PW determination during data analysis and how it can affect the CHON^+^ fragment identification.

## 2 Experimental Section

### 2.1 LToF-AMS

**Figure 1:**
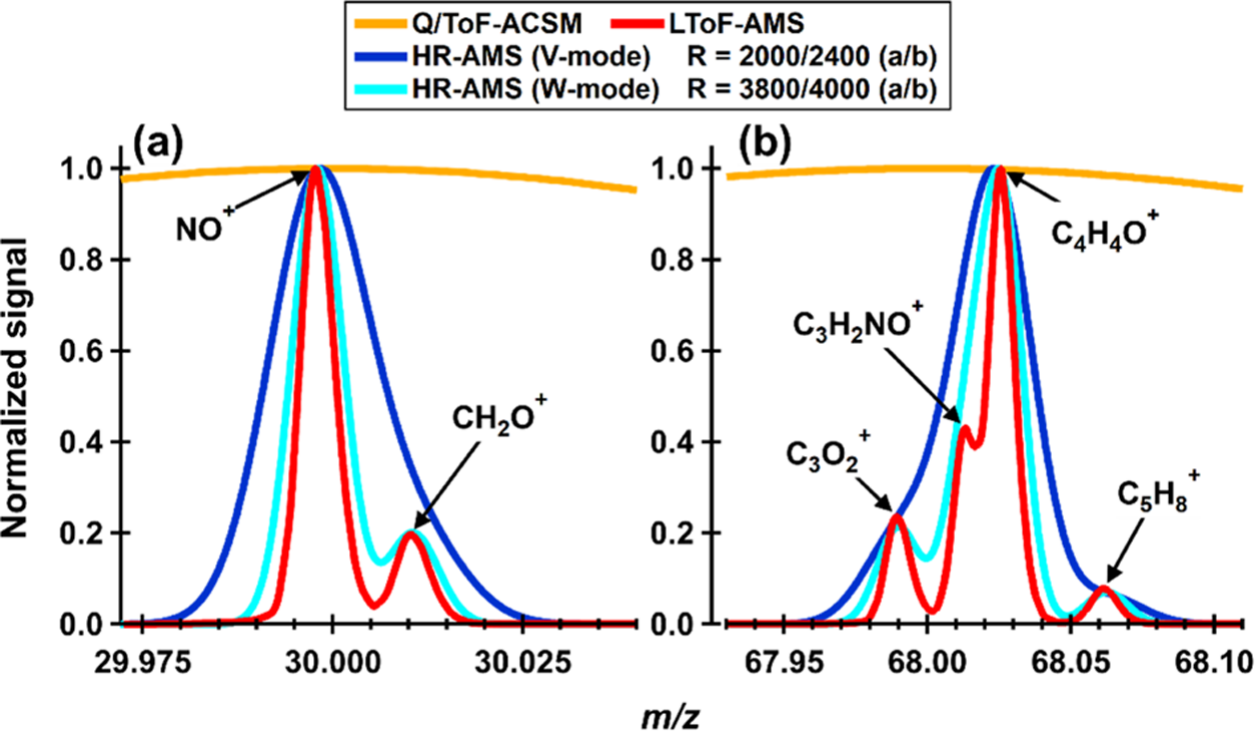
Comparison of the resolving power of different AMS versions. The LToF-AMS data are measured during our laboratory experiments, while the rest of the curves are produced artificially assuming the mass resolution of the instruments. At *m*/*z* 30 (a), the mass resolution for HR-AMS was set to 2000 and 3800 *M*/Δ*M* for V-mode and W-mode, respectively, and at *m*/*z* 68 (b), it was 2400 and 4000 *M*/Δ*M*, respectively. Q-ACSM and ToF-ACSM are UMR instruments.

The Aerodyne long time-of-flight Aerosol Mass Spectrometer (LToF-AMS) is a near-real-time instrument for measuring the size-resolved chemical composition of nonrefractory submicron aerosol (NR-PM_1_, where “nonrefractory” means that the AMS is only able to detect material that flash vaporizes at 600 °C). The LToF-AMS is similar to the high-resolution ToF-AMS (HR-ToF-AMS, hereafter HR-AMS^23^) but it is mounted with a longer ToF mass spectrometer chamber for increased mass resolution. The mass resolution of the LToF-AMS approaches 8000 *M*/Δ*M*, which allows further separation of close peaks in the mass spectrum.^27^ A comparison of the mass resolution for different versions of AMS is presented in Figure 1 in which the LToF-AMS curves are actual measurements from the laboratory while for the other instruments the curves are produced artificially following the mass resolution of each instrument version as reported in the literature. At *m*/*z* 30, the mass resolution for HR-AMS was set to 2000 and 3800, for V-mode and W-mode, respectively, and at *m*/*z* 68 it is 2400 and 4000, respectively.^23,27^ The quadrupole ACSM (Q-ACSM)^28^ and ToF-ACSM^29^ are unit mass resolution (UMR) instruments. At *m*/*z* 30 (Figure 1a), it is crucial to separate the organic fragment CH_2_O^+^ from NO^+^ for better total nitrate assessment as well as pON calculations (see Section 2.4 for details), The LToF-AMS and HR-AMS can clearly separate these two fragments. As the number of possible ions per unit mass quickly increases with mass, the higher mass resolution of the LToF-AMS becomes increasingly important at higher masses, for example, as seen for the separation of the C_3_H_2_NO^+^ fragment among the other ions at *m*/*z* 68 (Figure 1b).

#### 2.1.1 AMS Data Processing

The standard ToF-AMS data analysis is done within the analysis software SQUIRREL (for UMR analysis) and PIKA (for HR analysis) and is described in detail in previous studies.^23,30^ However, a brief summary of the *m*/*z* calibration and peak width function determination that are essential for HR analysis is presented here. The base for any HR analysis is the *m*/*z* calibration; with a poor *m*/*z* calibration, the user is not able to perform accurate peak identification, i.e., distinguishing for example C_3_H_2_NO^+^ from C_4_H_4_O^+^ as shown in Figure 1b. For the *m*/*z* calibration, only isolated ions that are present throughout the data should be used. The ions need to be isolated at full width at half-maximum (FWHM) but can have some nearby, low-signal, ions without affecting the *m*/*z* calibration. The chosen ions should be distributed throughout the whole *m*/*z* range where the HR analysis will be done. Common ions for the *m*/*z* calibration are air ions at *m*/*z* 28 (N_2_^+^), *m*/*z* 32 (O_2_^+^), and *m*/*z* 44 (CO_2_^+^) and three other internal background signals at *m*/*z* 149 (C_8_H_5_O_3_^+^), *m*/*z* 207 (C_5_H_15_O_3_Si_3_^+^), and *m*/*z* 279 (C_16_H_23_O_4_^+^). If sulfate is present, *m*/*z* 48 (SO^+^) and *m/z* 64 (SO_2_^+^) can also typically be used. In addition, organic ions can also be suitable as long as they meet the mentioned criteria. Furthermore, background signals from the filament (tungsten ions at *m*/*z* 91 (^183^W^2+^), *m*/*z* 182 (^182^W^+^), *m*/*z* 184 (^184^W^+^), and *m*/*z* 186 (^186^W^+^)) are also often utilized.

For determining the measured signal for each ion in high resolution, the peak width (PW) and peak shape (PS) must be defined. The PS is determined as the average peak shape of selected ions. The resolution is linked to the PW as (mass) resolution is defined as the mass of the peak divided by the FWHM. Thereby, a narrower PW corresponds to a higher resolution. In the AMS, PW is a function of *m*/*z* (PW increases with increasing *m*/*z*) and is determined by choosing ions throughout the *m*/*z* range (similar to the *m*/*z* calibration). Within PIKA, two separate PW fitting functions can be chosen; linear  or power law , where *a*, *b*, and *c* are parameters to be determined. This is done based on the PW of selected ions which should be isolated and present throughout the whole data set. Nearby overlapping ions can broaden the observed PW of a given ion, and if included in the calculations of the average PW, this can cause an incorrect PW function which ultimately affects the signal attribution during the HR fitting step (see Section 3.1).

In this study, we investigated the impact of including or omitting the W^+^ ions during the *m*/z calibration and PW determination, with emphasis on the PW. To this end, we analyzed the same data multiple times by including or omitting the W^+^ ions during the different data analysis steps. All other steps were performed in the same way for each iteration. The selection of suitable ions was determined by testing different combinations of ions until both the *m*/*z* calibration and PW reached the best possible accuracy.

### 2.2 Laboratory Measurements of pON

The laboratory experiments were done as part the Aerosol Chemical Monitor Calibration Center (ACMCC) pON experiments in 2018^31^ where the purpose was to compare simultaneously the response of different ACSM/AMS systems and to investigate the SOA physical properties and chemical composition formed from different pON precursors. Here, we focus only on the analysis of a subset of the data, collected by the LToF-AMS. As the whole experimental setup used during the ACMCC pON experiment is not relevant to this study, we will only give a brief description of the key parts used for this study (Figure S1).

SOA were generated in dry condition (relative humidity of 10%) and in the absence of any seeds in a Potential Aerosol Mass Oxidation Flow Reactor (PAM-OFR, hereafter PAM, Aerodyne Research, Inc.)^32,33^ by NO_3_ radical oxidation of single VOC precursors. Two biogenic monoterpenes (limonene and β-pinene) and one anthropogenic (guaiacol, typically emitted from anthropogenic (and natural) biomass burning) pON precursors were investigated. The pON precursors in the laboratory experiments were not chosen specifically to support the field measurements described in the next section, as the laboratory measurements were part of a broader project where the initial purpose was to compare simultaneously the response of different ACSM/AMS systems. All of the precursors studied have been selected as they were known to produce high yields of SOA from their NO_3_ oxidation.

NO_3_ radicals were produced through a continuous generation of dinitrogen pentoxide (N_2_O_5_) in the gas phase at room temperature (23 °C) using a laminar flow reactor (LFR) from NO_2_ + O_3_ and NO_2_ + NO_3_ reactions (OFR-iN_2_O_5_ method^34^). N_2_O_5_ injected into the PAM decomposes to generate NO_3_ and initiate the oxidation of VOCs.^34^ The O_3_ mixing ratio inside the LFR, [O_3_]_0,LFR_, was between 150 and 180 ppm (ozone analyzer, Model 202, 2B Technologies), and the [NO_2_]_0,LFR_/[O_3_]_0,LFR_ ratio was 2.0 for limonene experiments and 0.75 for β-pinene and guaiacol oxidation experiments. Direct monitoring of stable NO_3_/N_2_O_5_ generation was performed using an incoherent broad band cavity-enhanced absorption spectroscopy instrument (IBBCEAS).^35^ VOC (>98% purity, Alfa Aesar or Aldrich, diluted in ethanol at 50:50, v–v) were injected continuously using a microliter syringe pump (TriContinent C24000, 50 μL syringe) to reach stable initial concentrations into the PAM of about 710 ppbv for guaiacol and 1940 ppbv for both monoterpenes. In such conditions, NO_3_ concentrations in the PAM were about 1–5 ppbv inducing NO_3_ exposure of about 8 × 10^13^ molecules cm^–3^ corresponding to about 2 nights of aging.^34^ The produced, polydisperse pON at constant concentrations were then size-selected (200, 300, and 400 nm for guaiacol, limonene, and β-pinene SOA, respectively) using an aerodynamic aerosol classifier (AAC, Cambustion^36^) and monitored using a scanning mobility particle sizer (SMPS, TSI, DMA 3080, CPC 3776). The monodisperse aerosol, at different concentration levels obtained using a “dilution loop” made with a total filter regulation setup (0.3 μm; TSI), was analyzed by the LToF-AMS with 1 min time resolution.

AMS data from the ACMCC pON experiment were analyzed with the standard ToF-AMS Analysis software packages SQUIRREL (version 1.63H) and PIKA (version 1.23H) within Igor Pro (version 6.37 and 8.04, WaveMetrics Inc). For the most part, we processed the data with normal AMS methods, but we paid extra attention to both *m*/*z* calibration and PW determination. When performing the peak identification during the HR analysis, we applied a limit of acceptable fractional residual of 0.05. The residual within PIKA describes the difference between the measured signal and fitted ions as a fraction of peak height. In practice, this means that if the residual was over 0.05, we assumed there was a relevant ion missing at that *m*/*z* and added one more ion to be fit (see Section 3.1 for details). Once the residual was lower than 0.05, no more ions were fitted.

The ionization efficiency (IE) calibrations were performed with monodisperse ammonium nitrate particles on site and we used a collection efficiency of 1 for these experiments, as the absolute aerosol loadings were not of importance for our analysis.

### 2.3 Field Measurements of pON at SMEAR II

The ambient measurements were conducted in Hyytiälä, Finland, at the SMEAR II station (61°51′ N, 24°17′ E, 181 m above sea level^37^). SMEAR II is a well-known atmospheric measurement supersite focusing on tracking the exchange of matter, energy, and momentum between the biosphere and atmosphere. The measurement site is located within the boreal forest with only minor nearby anthropogenic sources apart from two sawmills located ca. 7 km southeast of the station.^38^ Depending on the wind directions, the sawmills are a considerable source of monoterpenes and SOA^26,38,39^ (also discussed in Section 3.3).

In this study, we will focus on ambient data obtained between April 8 and May 4, 2016, by the LToF-AMS. The same LToF-AMS instrument was used later in the laboratory experiments described above. The LToF-AMS sampled from the same inlet line as an ACSM that is part of the SMEAR II long-term measurements.^26^ The LToF-AMS was located in an air-conditioned container, with the sampling done through the roof of the container through a PM_2.5_ cyclone. A Nafion dryer in the sampling line kept the relative humidity below 30%. The sampling flow rate was set to 3 L min^–1^ up until the instrument and the LToF-AMS sampled at 0.1 L min^–1^ through its critical orifice. The AMS was operated with a 3 min time resolution. The original data were averaged to 30 min for the analysis in this study to improve the signal-to-noise ratios. Ionization efficiency calibrations were performed with dried and size-selected ammonium nitrate particles during the campaign. We applied a constant collection efficiency (CE) factor of 0.5 when calculating the particle mass concentration.

For the SMEAR II AMS data analysis, the software package versions were SQUIRREL 1.62A and PIKA 1.22A. The data analysis was done with Igor Pro (version 6.37 and 8.04, WaveMetrics, Inc.).

### 2.4 pON Quantification through AMS Measurements

First, as we will use several acronyms for particulate organic nitrate-related variables, below is a description of the relevant terminology used in the Results and Discussion section:

- NO_3_ (nitrate): total nitrate mass concentration measured by the AMS.
- Org (organic): total mass concentration of organics measured by the AMS.
- pON (particulate organic nitrate): mass concentration of pON (pON = pON_NO_3__+ pON_Org_).
- pON_NO_3__: mass concentration of the nitrate group of pON.
- pON_Org_: mass concentration of organic part of pON.
- frac_pON,NO_3__: fraction of pON_NO_3__ to total NO_3_ (eq 1).

To estimate pON at SMEAR II from AMS data, we applied the following formula that gives the fraction of organic nitrate (frac_pON,NO_3__) from the total measured nitrate using the NO^+^/NO_2_^+^ ratio measured by the AMS^21^
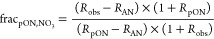
1Here, *R*_obs_ is the observed NO^+^/NO_2_^+^ ratio in the sample of interest, *R*_AN_ is the ratio measured during AN calibrations, and *R*_pON_ is the NO^+^/NO_2_^+^ ratio for pure pON. As in previous studies,^12,16,40^ we assumed *R*_pON_ = 10. We additionally note that *R*_pON_ values for pON generated from NO_3_ oxidation of α-pinene + NO_3_ range from 8.42 to 11^20,41^ and that α-pinene was the most abundant monoterpene at SMEAR II.^42,43^ The choice of *R*_pON_ = 10 is further motivated in Section 3.3.1 by Figure 7. By multiplying frac_pON,NO_3__ with the total measured nitrate (NO_3_), we get the mass concentration of nitrate in pON, i.e., pON_NO_3__.

Due to the high mass resolving power of the LToF-AMS, both NO^+^ and NO_2_^+^ can unambiguously be resolved from interferences at unit mass *m*/*z* 30 (CH_2_O^+^) and *m*/*z* 46 (CH_2_O_2_^+^). This is especially important at SMEAR II as the organic fragments constitute a large fraction of the signal at their unit mass and are occasionally even larger than the nitrate fragments. The mass concentration of total particulate organic nitrate (pON) can be estimated by assuming a molecular weight for the pON (MW_pON_; eq 2). Previous studies^10,12,16,19,21,40^ have assumed the MW of pON between 200 and 300 g mol^–1^. However, we used MW_pON_ = 265 g mol^–1^ (with 200 and 330 g mol^–1^ as lower and upper limits of MW_pON_, respectively) based on earlier FIGAERO-CIMS measurements conducted at SMEAR II during the spring of 2014.^15^
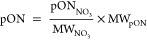
2

## 3 Results and Discussion

We start this section by discussing the potential of the LToF-AMS for pON detection using both the laboratory data gathered during the ACMCC pON experiment and the ambient SMEAR II data and evaluate how CHON^+^ quantification is affected if W^+^ ions were incorporated in the peak width determination for high-resolution peak fitting. We then present results from the SMEAR II field campaign, with emphasis on the contribution of pON to the total NR-PM_1_ and its diurnal behavior.

### 3.1 Utilization of Tungsten (W^+^) Signals for AMS Peak Width (PW) Function

Tungsten ions (W^+^) are part of the AMS mass spectra. They originate from the AMS filament and are typically considered as default peaks for mass calibration, and potentially even for the PW function determination. We tested how the use of these peaks affects the HR results by showing examples of how CHON^+^ fragments can be affected.

**Figure 2:**
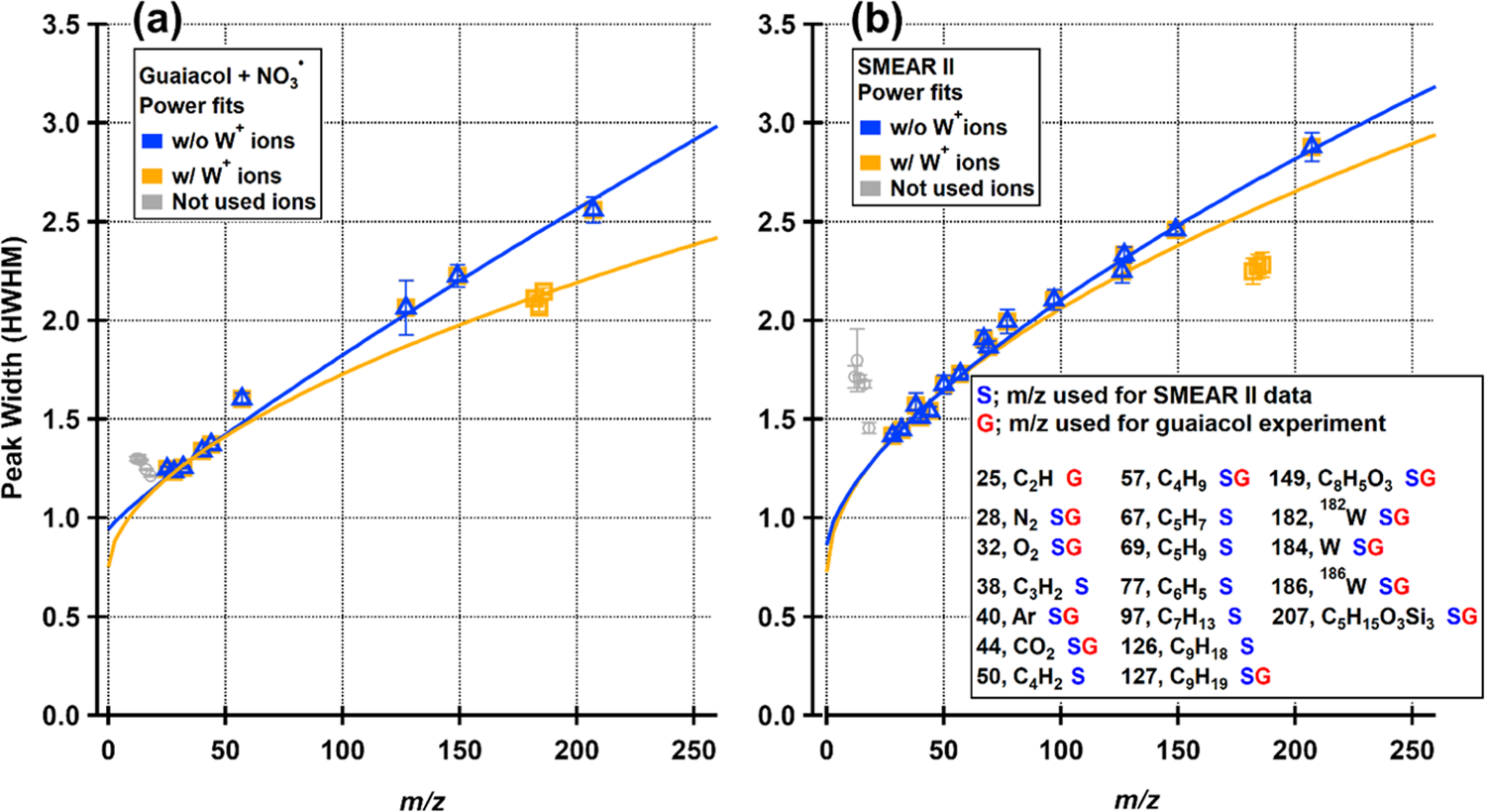
Peak width (PW) functions for ACMCC (guaiacol SOA) (a) and SMEAR II data (b). Blue and yellow markers are from the data processing scenario when no W^+^ ions were used and when W^+^ ions were incorporated, respectively. The gray markers show disqualified ions at low *m*/*z* (<20) and are C (*m*/*z* 12), CH (*m*/*z* 13), N (*m*/*z* 14), O (*m*/*z* 16), and H_2_O (*m*/*z* 18).

The difference in the PW functions when W^+^ ions are utilized and omitted for both SMEAR II and ACMCC (guaiacol SOA) data is presented in Figure 2. The ions chosen for the PW functions are listed in the textbox of Figure 2. The ions are chosen as described in Section 2.1.1. For ions *m*/*z* < 20, the PW does not follow the general PW trend and these ions are not included in the fit. This behavior is characteristic for the LToF-AMS and cannot be tuned out. It is clear that the W^+^ ions do not follow the PW function and have a narrower PW than the rest of the ions. This suggests that the W^+^ ions have a narrower energy distribution than the ions from aerosol particles, and may be related to the differences in the source regions of the ions. The resistively heated tungsten filament is the source of the 70 eV electrons as well as the W^+^ ions, while the sample ions are ionized in a region in front of the vaporizer, after interaction with the electrons. This may cause the sample ions to enter the guiding ion optics with a larger variation in energies, which causes their flight times in the ToF chamber to vary more than for the W^+^ ions. When W^+^ ions are used for the ACMCC pON experiment data (guaiacol SOA), the PW functions determined with W^+^ and without W^+^ start to diverge already around *m*/*z* 70. The difference increases as a function of *m*/*z* (Figure 2a). For the SMEAR II data set (Figure 2b), the two scenarios start to diverge later: the absolute differences in PW functions observed at *m*/*z* 70 and *m*/*z* 100 for the ACMCC guaiacol data set are reached at *m*/*z* 90 and *m*/*z* 145, respectively, for the SMEAR II data. This big difference between the ACMCC guaiacol and SMEAR II data sets is explained by the number of ions used for the PW determination. Only 9 ions were found to be sufficiently isolated for the ACMCC guaiacol data set when W^+^ peaks were omitted, while 15 ions were found for the SMEAR II data set. As the W^+^ ions are at high *m*/*z* (182, 184, 186), they affect the PW function more in the case when fewer other ions are present at high masses. In the SMEAR II data set, more suitable ions were found at *m*/*z* > 50, including two ion signals at *m*/*z* 97 and 126, due to more diverse sources. We also tested including only one W^+^ ion (at *m*/*z* 184) for the PW function for the SMEAR data, but interestingly, the result did not significantly differ from the case where all three W^+^ ions were used (under 2% difference in the PW function at *m*/*z* 184).

The stability of the ions used for the PW function was ensured from the time series of the individual ions (time series for the SMEAR II data is shown in Figure S2). All ions are stable throughout the whole data set and the signal is not contaminated with nearby ions that would broaden the PW or have some unusual time-dependent behavior.

**Figure 3:**
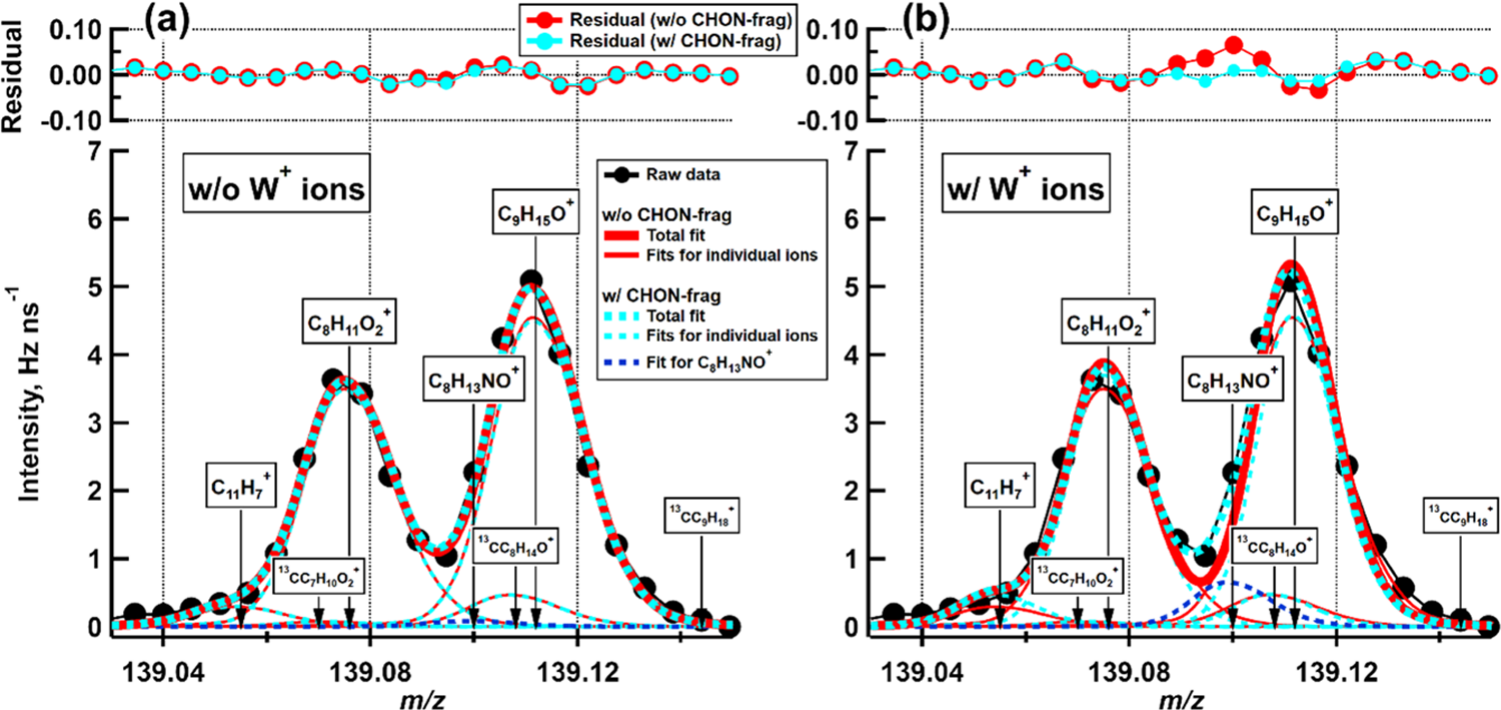
HR-peak fitting at *m*/*z* 139 for limonene + NO_3_ radical reaction from the ACMCC pON experiment. In each panel, red lines show the individual (thin) and total (thick) fits for the case when the ion C_8_H_13_NO^+^ was excluded from the fitting and blue dashed lines show the same, except that the organic nitrate ion was included in the fit. (a) Fitting when W^+^ ions were not used during PW determination; (b) fitting when W^+^ ions were included in the PW determination.

The effect of W^+^ incorporation in the PW determination may seem small but will have a considerable impact on, e.g., CHON^+^ fragment identification during HR peak fitting. This is illustrated in Figure 3, where the ACMCC (limonene SOA) data has been processed in an identical way except for the usage of W^+^ ions during the PW determination. In this example, we fit four ions at *m*/*z* 139, in addition to three isotopes with magnitude determined by the parent ion at *m*/*z* 138. In Figure 3a, where no W^+^ ions are used, the signal from the C_8_H_13_NO^+^ ion (dark blue dashed line) is negligible: the residual of the case where the ion is fitted does not significantly differ from the case when it is not fitted. Figure 3b represents the scenario where W^+^ ions were used for PW determination, and we expect the PW to be narrower than it should be. Now the same C_8_H_13_NO^+^ ion is significantly contributing to the sum fit.

A few data sets from ACMCC (guaiacol/limonene/β-pinene + NO_3_ radicals) were analyzed twice with the two data processing scenarios and we applied the residual limit of 0.05 (as described in Section 2.2) for all of these data sets. The difference in the mass spectra of the two data processing scenarios is presented in Figure S3. For example, in the limonene + NO_3_ radical case (Figure S3b), the number of CHON^+^ fragments fit in the W^+^ free scenario was 9, while in the W^+^ incorporated scenario, 25 CHON^+^ fragments were fit. While the relative difference is substantial, despite the doubling of the number of ions fitted, their contribution to the total organic mass was still less than 1% (Figure S4). For instance, the contribution of CHO_>1_N^+^ fragments for limonene + NO_3_ radical increased almost by a factor of 5. This makes CHO_>1_N^+^ fragments contribution increase from 0.064 to 0.30% when comparing the mass contribution against the data processing scenarios performed without W^+^.

The major organic families, CH^+^, CHO_1_^+^ (and CHO_>1_^+^ for β-pinene) contribute each >10% and altogether >90% to the total organic mass. For these, the mass fraction differences between the two data processing scenarios are minor (<1%). The number of ion fits within these families were the same for the two cases with a few exceptions. Therefore, we conclude that the potential error in PW caused by including the W^+^ ions may often go unnoticed, especially if analysis is only focused on the largest signals. The largest effects are for small signals, and one of the major risks comes if some of these signals are used as a marker, e.g., looking for CHON^+^ fragments as tracers for pON would be relevant for our study.

### 3.2 NO^+^/NO_2_^+^ Ratios and Mass Spectral Differences during the ACMCC pON Experiment

It should be noted that the following discussion concerns data that was processed without the utilization of W^+^ ions for the PW function as W^+^ ions clearly do not represent the PW for the rest of the ions, as described in the previous sections.

**Figure 4:**
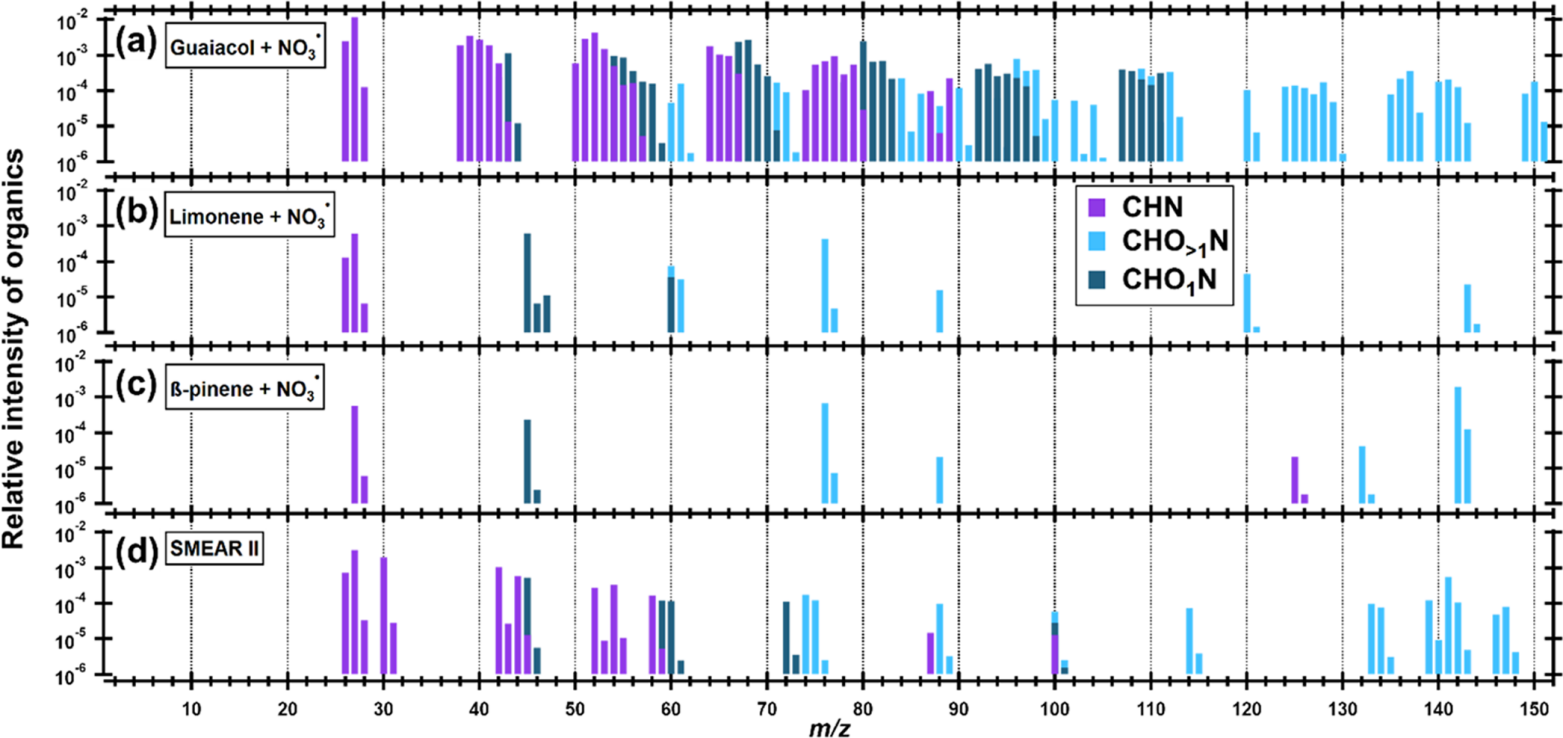
Mass spectra of CHN^+^ and CHON^+^ fragments from the ACMCC pON expriments and SMEAR II. (a) Guaiacol + NO_3_ radical experiment, (b) limonene + NO_3_ radical experiment, (c) β-pinene + NO_3_ radical experiment, and (d) SMEAR II mass spectra. Note: logarithmic *y*-axis.

The measured NO^+^/NO_2_^+^ ratios for SOA generated from NO_3_ radical oxidation of guaiacol, limonene, and β-pinene were 6.60, 5.96, and 6.23, respectively. These results are at the lower end of the range of 5–15 previously measured.^20,41,44−49^ The CHN^+^ and CHON^+^ (including both CHO_1_N^+^ and CHO_>1_N^+^) ions fitted in the mass spectra of the SOA sampled by the LToF-AMS from the different pON precursors are presented in Figure 4 (the complete mass spectra are presented in Figure S5). While the guaiacol SOA has both CHN^+^ and CHON^+^ fragments spread across the whole *m*/*z* axis, the limonene and β-pinene SOA have only a few sporadic fragments. There is also a large difference in the number of fitted CHON^+^ fragments for the different data sets: 72, 9, and 5 CHON^+^ fragments were fitted for the guaiacol, limonene, and β-pinene data sets, respectively. All of the fitted CHON^+^ ions for the ACMCC pON experiment (and SMEAR II) data sets are presented in Table S1.

Precursor-specific CHON^+^ ions included C_3_H_6_NO_4_^+^ (*m*/*z* 120) and C_7_H_13_NO_2_^+^ (*m*/*z* 143) in limonene SOA and C_4_H_6_NO_4_^+^ (*m*/*z* 132) in β-pinene SOA. We note that 67 of the 72 CHON^+^ ions were unique for guaiacol SOA; CHON^+^ ions with the largest signals included C_2_H_0-4_NO^+^ (*m*/*z* 54–58), C_3_H_1-2_NO^+^ (*m*/*z* 67-68), and C_4_H_2-3_NO^+^ (*m*/*z* 80-81). Figure S6 shows example HR spectra of C_2_H_2_NO^+^, C_3_H_2_NO^+^, CH_2_NO_3_^+^, and C_3_H_6_NO_4_^+^ ion signals. This demonstrates also how the resolution of the LToF-AMS can be utilized in detecting CHON^+^ fragments.

### 3.3 Overview of the LToF-AMS Measurements at SMEAR II

**Figure 5:**
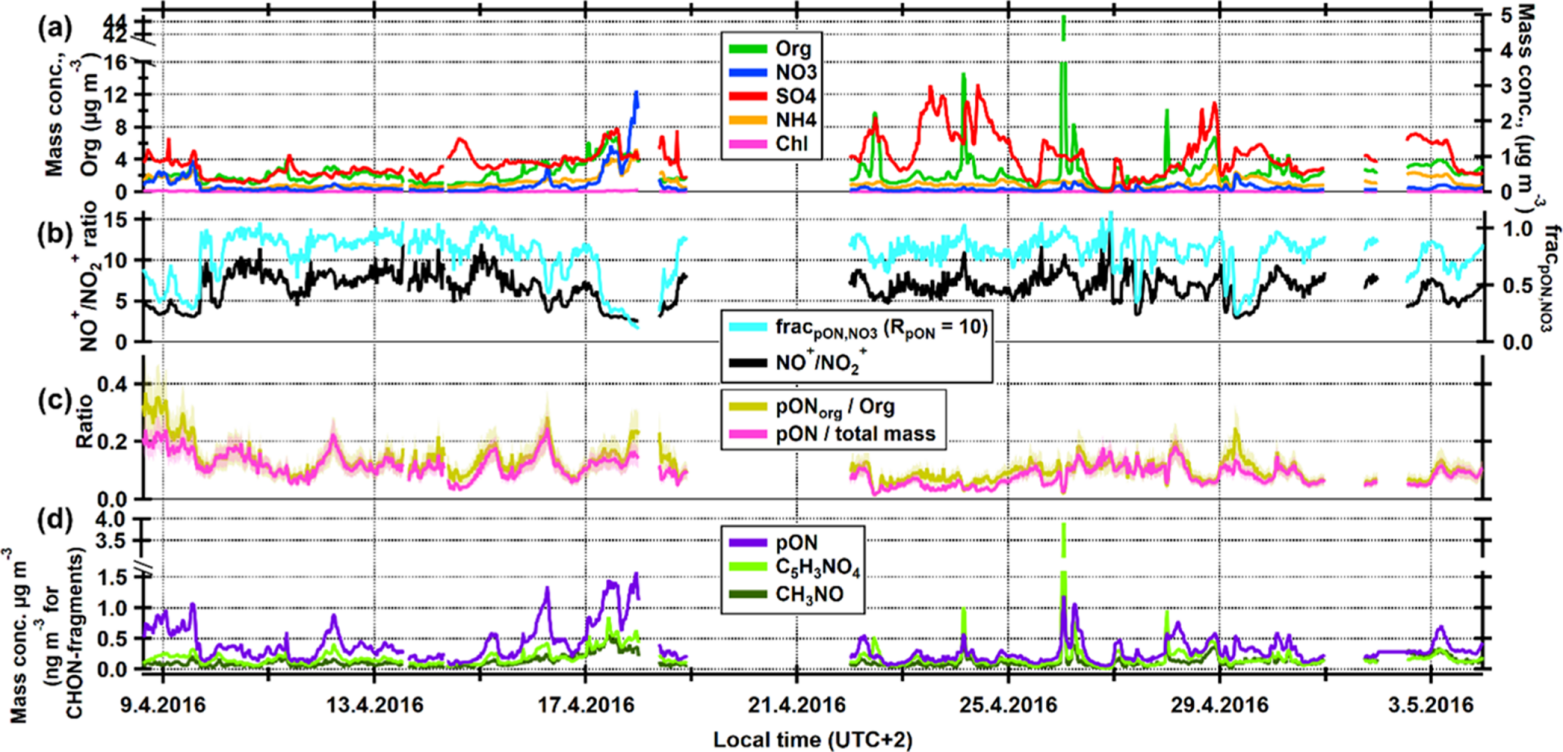
SMEAR II data with time series of (a) chemical species; (b) NO^+^/NO_2_^+^ ratio and frac_pON,NO_3__; (c) pON/total mass and pON_org_/Org; and (d) pON, C_5_H_3_NO_4_^+^, and CH_3_NO^+^ ion fragments. In (a), the mass concentration of organics is shown on the left *y*-axis and the rest of the chemical species are shown on the right *y*-axis. In (c), solid lines represent values calculated with MW_pON_ = 265 g mol^–1^ and the shaded areas are calculated with MW_pON_ 200 and 330 g mol^–1^. Note that in (d), the units are μg m^–3^ for pON, but ng m^–3^ for the CHON^+^ ions fragments.

The median NR-PM_1_ concentration was 3.3 μg m^–3^ (2.3 and 4.3 μg m^–3^ as the 25th and 75th percentiles) during the ambient measurement period (from April 8 to May 5, 2016) at SMEAR II. The median mass concentrations for organics, nitrate, sulfate, ammonium, and chloride were 2.0, 0.081, 0.81, 0.20, and 0.0067 μg m^–3^, respectively. The time series of the submicron chemical components are shown in Figure 5a. Based on wind direction analyses, the exceptionally high plume of organics (over 40 μg m^–3^ of Org) detected the night between April 25 and 26 (1.5 h of data), most likely originates from the nearby sawmills. Therefore, we excluded this plume (3 data points, the highest Org signal in Figure 5) from all Pearson’s *r*^2^ correlation coefficient calculations as they would control the calculated *r*^2^ values; for example, the *r*^2^ for NO_3_ vs C_5_H_3_NO_4_^+^ (in Figure 8d) increases from 0.36 to 0.60 if the plume data points are included.

**Figure 6:**
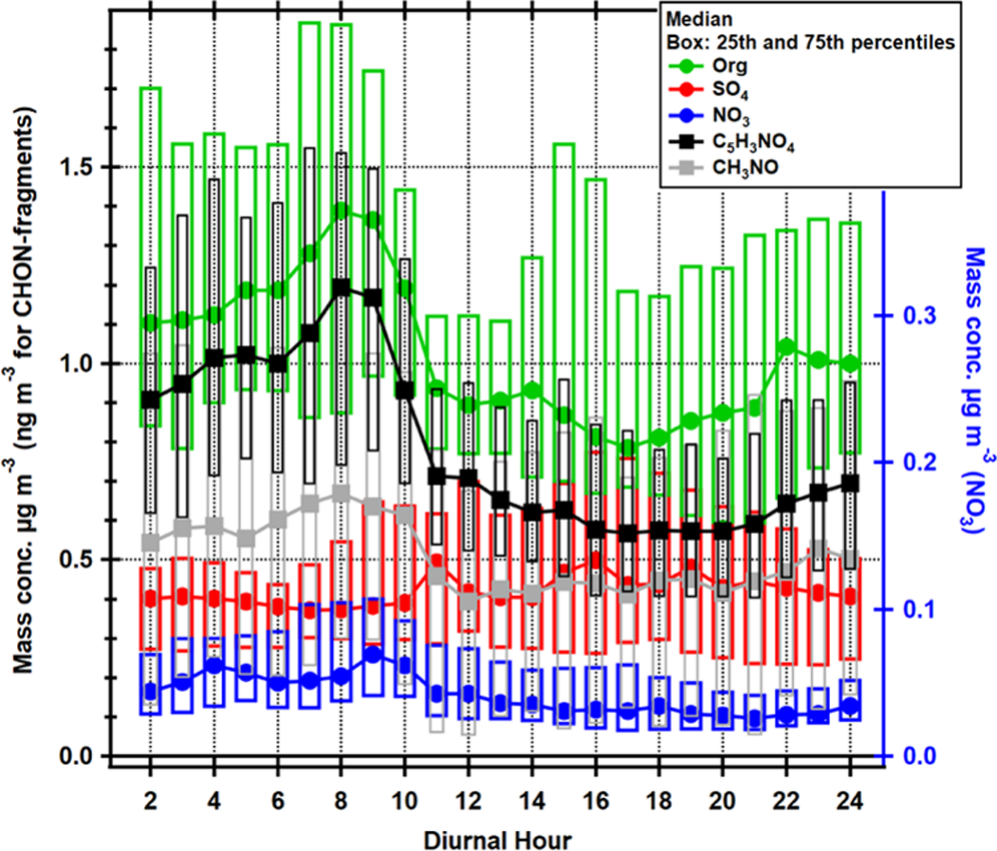
Diurnal cycles of Org, NO_3_, SO_4_, C_5_H_3_NO_4_^+^, and CH_3_NO^+^ ion fragments. The markers show the hourly median values, and boxes are drawn between the 25th and 75th percentiles. The *x*-axis represents the local time of day (UTC + 2).

The diel trends of Org, NO_3_, and SO_4_ are shown in Figure 6. Both Org and NO_3_ have maxima during the night and early morning while SO_4_ does not have a clear diel trend. These diel trends are in line with the long-term measurement data of NR-PM_1_ species.^26^ The diel trend of the two CHON^+^ fragments in the same graph is discussed in more detail in the next section. Unfortunately, we lack monoterpene measurements during the measurement period and are therefore not able to deduce the main drivers behind the (pON-related) diel trends. However, the diel trends of monoterpenes at SMEAR II are quite well known and are largely driven by the boundary layer height, with below-canopy concentrations peaking at night despite emissions peaking during the day.^50^ Therefore, we can only draw some general conclusions from our data.

#### 3.3.1 Particulate Organic Nitrate

**Figure 7:**
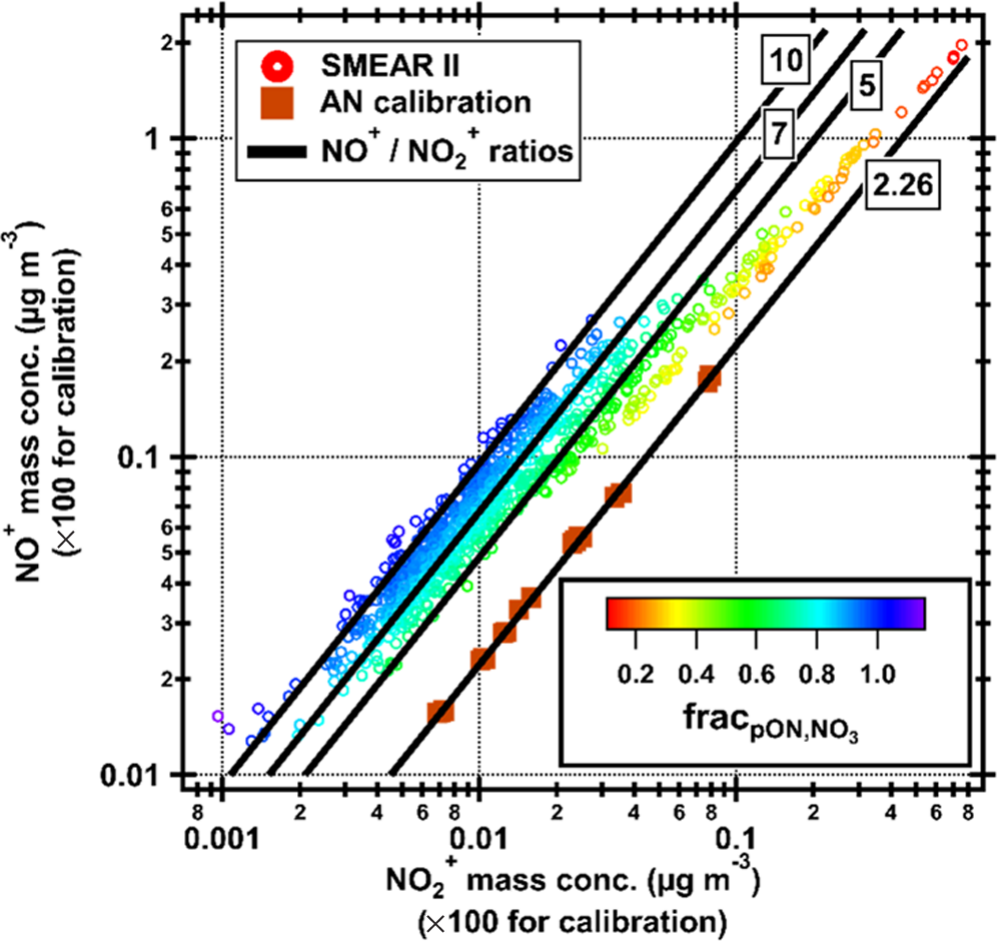
NO^+^ vs NO_2_^+^ mass concentration at SMEAR II. Circles are ambient data with a color scale that shows frac_pON,NO_3__. Squares are from an ammonium nitrate (AN) calibration during the measurement period (divided by 100 for easier comparison with the low ambient concentrations). The lines represent different NO^+^/NO_2_^+^ ratios where the 2.26 line is fitted to the AN calibration data.

Measured *R*_obs_ values ranged between 5 and 10 (median 6.8) and dropped below 5 only under higher ammonium nitrate influence (Figure 5b, below 5, e.g., on April 9th, 16th, 17th, and 29th), suggesting that the typical background NO_3_ is almost solely organic. Figure 7 displays the NO^+^ vs NO_2_^+^ of both ambient and AN calibration data. The black lines in the figure represent NO^+^/NO_2_^+^ ratios of 2.26, 5, 7, and 10, where the NO^+^/NO_2_^+^ = 2.26 line was measured during AN calibration. As seen, the NO^+^/NO_2_^+^ = 10 line fits the outer edge of the data well, with only a few points above the line. This line would represent a pure pON event, and a lower *R*_pON_ would clearly overestimate frac_pON,NO_3__. Using *R*_pON_ = 7, frac_pON,NO_3__ would repeatedly give unphysical values above one, indicating that 7 is a too low value for SMEAR II using our instrument. It can also be noticed that only a few points are close to the AN calibration line, further indicating that NO_3_ at SMEAR II is almost never purely inorganic AN.

The fraction of total NO_3_ that was found in pON (frac_pON,NO_3__, calculated with eq 1) is shown in Figure 5b and had a median value of 0.83. The separation of NO^+^ and NO_2_^+^ from the organic fragments at the same unit mass at Hyytiälä is crucial as interference of these organic fragments can affect the pON concentrations calculated by the NO^+^/NO_2_^+^ ratio. The median ratio of CH_2_O^+^/NO^+^ (at *m*/*z* 30) is 0.43, and that of CH_2_O_2_^+^/ NO_2_^+^ (at *m*/*z* 46) is 0.42. Regardless of the large variation in these ratios (Figure S7a), the median frac_pON,NO_3__, calculated by the UMR ratio of *m*/*z* 30 and *m*/*z* 46 (a proxy for the NO^+^/NO_2_^+^ ratio, in the case that only UMR data from an AMS/ACSM is available^26^), is only 2% higher than that calculated by the NO^+^/NO_2_^+^ ratio (Figure S7b). Nevertheless, the UMR calculations can differ up to ±40% from the HR calculations (Figure S7c).

Figure 5c shows the estimated fraction of pON to the total mass to be 9.7% (median, with 6.4 and 12% as the 25th and 75th percentiles, respectively) while the pON_org_ to Org was 11% (median, with 8.3 and 14 as the 25th and 75th percentiles, respectively), which is in line with previous pON quantifications from SMEAR II.^15,51^ The median pON mass concentration was 0.32 μg m^–3^ (0.20 and 0.69 μg m^–3^ as the 25th and 75th percentiles), as shown in Figure 5d.

**Figure 8:**
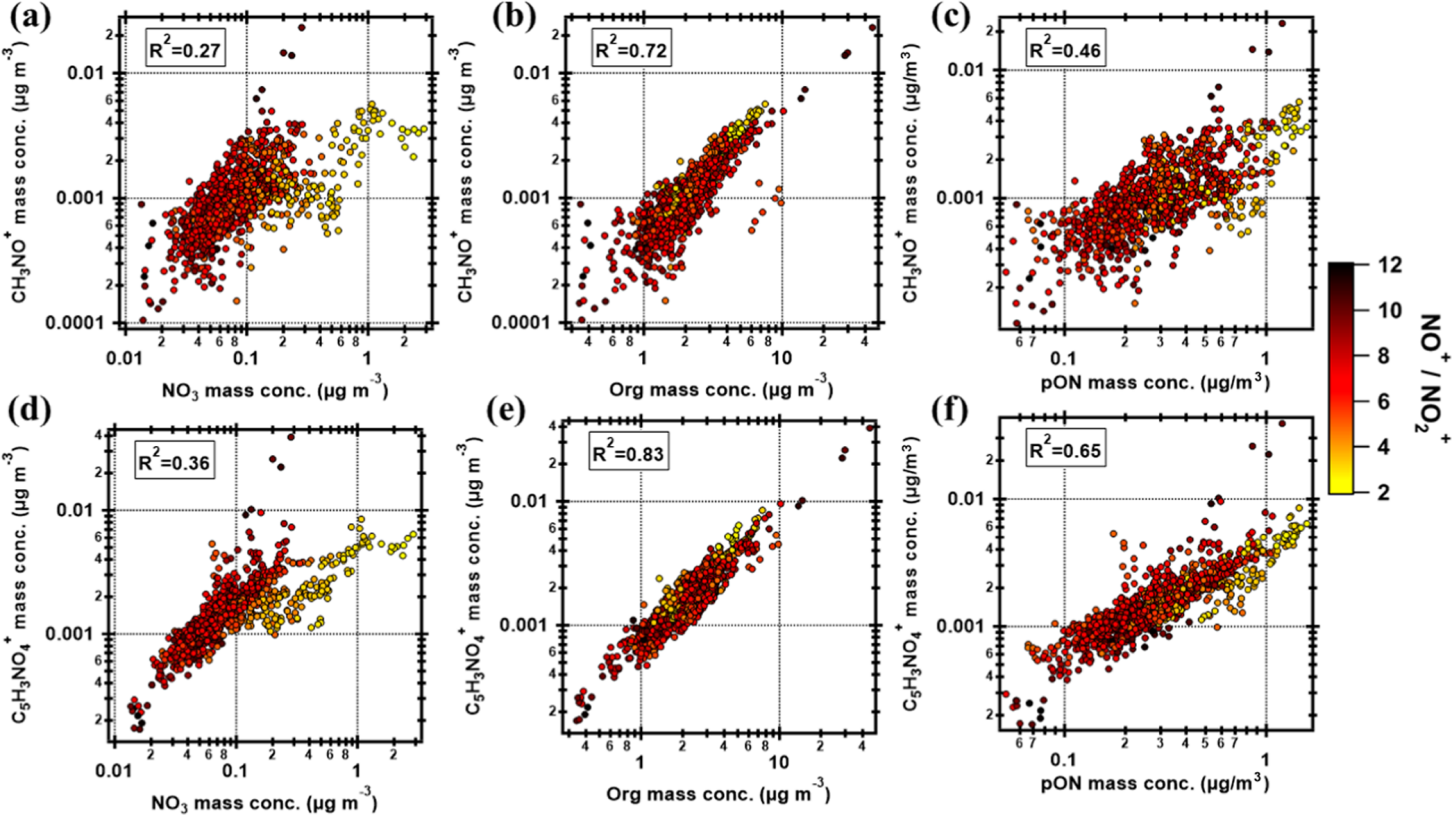
Scatter plots between CH_3_NO^+^ and C_5_H_3_NO_4_^+^ ion fragments against NO_3_, Org, and pON at SMEAR II. The color scale shows the NO^+^/NO_2_^+^ ratios in which yellow colors indicate the presence of inorganic ammonium nitrate. The Pearson correlation coefficients (squared) are shown in each subplot. Note that the data are displayed in log–log scales.

From the HR analysis, we identified 18 CHON^+^ fragments (Table S1) in ambient pON, which together explain 0.3% of the total organic signal. The majority of these fragments (>65%) are not detected in any of the ACMCC pON experiments and are presented, together with the CHN^+^ fragments, in Figure 4d. The two most abundant were C_5_H_3_NO_4_^+^ (*m*/*z* 141) and CH_3_NO^**+**^ (*m*/*z* 45) (Figure S8 for HR fits during the data analysis and time series in Figure 5d). The former is somewhat surprising, as a single large CHON^+^ signal at high mass, but we could not find any potential other ion that would be close enough in mass to explain the signal at *m*/*z* 141. Both CH_3_NO^+^ and C_5_H_3_NO_4_^+^ correlate well with Org (Figure 8b,e) and with pON (Figure 8c,f), although Org did not correlate well with pON (Pearson *r*^2^ is 0.38, Figure S9). The Pearson *r*^2^ are 0.72 and 0.83, respectively, between the fragments and Org and 0.46 and 0.65, respectively, between the fragments and pON, while the Pearson *r*^*2*^ between the fragments and NO_3_ are reduced to 0.27 and 0.36, respectively (Figure 8a,d). It is somewhat surprising that both CHON^+^ fragments correlate better with Org than pON, but it could be related to the way Org and pON are calculated. Org is the sum of many directly measured ions, while pON is calculated based on only a few measured signals and an *R*_pON_ with some uncertainty (eqs 1 and 2). This can lead to more scatter (and therefore worse correlation) for pON against separately measured CHON^+^ ions. In any case, this result indicates that either the pON concentrations are quite uncertain, or that the CHON^+^ fragments are not good representatives of total pON.

As expected, the CHON^+^ vs NO_3_ plots (Figure 8a,d) show most scatter at low NO^+^/NO_2_^+^ values (i.e., AN-dominated scenarios), again indicating the organic origin of NO_3_ most of the time. This is consistent with the high frac_pON,NO_3__ values corresponding to dominant organic NO_3_ during this measurement period. The three points excluded from the Pearson’s *r*^2^ calculations (from the nearby sawmill) also showed the highest concentrations of CH_3_NO^+^ and C_5_H_3_NO_4_^+^. Previous HR-AMS measurements at SMEAR II during spring 2011^14^ concluded that the highest pON concentrations arose from sawmill plumes like the one we detected.

CH_3_NO^+^ and C_5_H_3_NO_4_^+^ also showed a diel trend with maximum right after sunrise and minimum before sunset (Figure 6), clearly following the Org diel trend. The fast drop in both CHON^+^ fragments and Org during morning hours is a strong indicator that the boundary layer height plays a strong role in the diel trend. Furthermore, the pON_org_/Org and pON/total mass showed a similar trend with maximum (median 0.14 and 0.13) after sunrise and minimum (median 0.081 and 0.064) before sunset (Figure S10). Unlike the CHON^+^ fragments and Org, the diel trends of the ratios are less sharp, following the temperature inversely quite well (Figure S11, decreasing ratio values with increasing temperature), suggesting that volatility may play a role as well, with pON on average being more volatile than non-nitrated organics. Previous pON measurements from SMEAR II during spring of 2014 reported a maximum and minimum value for pON/Org as 0.35 and 0.15, while our corresponding values are 0.18 and 0.11.^15^ The difference may be due to inter-annual variability or different sets of instruments used in the studies. As both measurement campaigns were relatively short, they do not give an accurate climatological overview of pON mass fractions at SMEAR II. In addition, the diel trends of NO*_x_*, NO, and O_3_ (Figure S12) are similar during our measurement as they were in the previous campaign.

During the ACMCC pON experiments, CH_3_NO^+^ was detected in the limonene and β-pinene SOA, while C_5_H_3_NO_4_^+^ was detected in guaiacol SOA. As both limonene and β-pinene are detected at SMEAR II^42,52^ and have biogenic origin, they, along with other BVOCs (e.g., α-pinene), are potential precursors for the pON detected at the site. As guaiacol is a biomass burning tracer^53−55^ and SMEAR II is known for low biomass burning organic aerosol (BBOA),^56^ it is not expected that the biggest CHON^+^ fragment would be related to biomass burning, in particular as it tracked the total organic loading very well throughout the measurement period. Therefore, although C_5_H_3_NO_4_^+^ is detected at SMEAR II, we do not think it is a good marker ion for guaiacol-nitrated SOA. Overall, the good correlation of the observed CHON^+^ fragments with organics (and pON) means that they are not suitable as markers for different types of pON observed during the measurements presented here. However, at sites with intermittent contributions from biomass burning or other types of organic aerosol, CHON^+^ markers may still provide some useful insights. Further studies in suitable locations are needed to answer these questions.

## 4 Conclusions

We conducted both ambient and laboratory measurements with an LToF-AMS to study pON and the capability of the LToF-AMS to resolve CHON^+^ ions. As the pON molecules cannot directly be measured by an AMS, due to fragmentation, one needs to take into consideration the possible sources of error during the data analysis when calculating pON concentration from AMS data. Using the high resolution of the LToF-AMS, we were able to unambiguously differentiate NO^+^ from CH_2_O^+^ at *m*/*z* 30 and NO_2_^+^ from CH_2_O_2_^+^ at *m*/*z* 46, which is needed when using the NO^+^/NO_2_^+^ ratio to estimate the fraction of organic nitrate from the total nitrate. This separation is crucial, especially as our field measurements were conducted at SMEAR II where the organic fragments at *m*/*z* 30 and *m*/*z* 46 are large and, occasionally, even bigger than the nitrate fragments. As the long-term measurements of NR-PM_1_ at SMEAR II are conducted by a Q-ACSM,^26^ this mass spectral behavior should be taken into account if one wants to use UMR data to estimate pON at the site.

Our measurements suggest that pON (including both the organic and nitrate part) accounts for about 10% of both the total NR-PM_1_ mass and organics at SMEAR II during springtime and that the background level of NO_3_ is almost solely organic. There was also a clear diel trend with maximum in early mornings for pON fragments and the fraction of pON to total aerosol mass. Our results are in line with previous studies at SMEAR II.^14,15,51^ Another study at SMEAR II during Sept 2016^57^ reported alkyl nitrate formation from reactions of monoterpene and NO_3_ radicals both during night and day with a lifetime of approximately 2 h for these gas phase species. In addition, more particle phase, compared to gas phase, organic nitrate compounds with a clear nighttime diel trend were found at SMEAR II during the spring of 2014.^15^ These two studies support our findings for pON formation at SMEAR II and suggest that BVOC + NO_3_ radical chemistry, producing gas phase organic nitrates that are efficiently transferred to the particle phase, plays an important role in SOA formation at SMEAR II. Furthermore, the importance of NO_3_ radical chemistry is supported by Peräkylä et al.^50^ and Liebmann et al.,^58^ where the highest NO_3_ radical concentrations and reactivities at SMEAR II are reported to take place during early mornings and nights.

In addition to the field measurements at SMEAR II, we conducted laboratory measurements to study the response of the LToF-AMS to SOA produced from NO_3_ radical oxidation of three different VOCs (guaiacol, limonene, and β-pinene). The NO^+^/NO_2_^+^ ratio from the laboratory measurements was lower compared to the NO^+^/NO_2_^+^ ratio observed at SMEAR II, but the SMEAR II observations were closer to previously reported values of pON from reactions between NO_3_ radicals and α-pinene, which is the most abundant monoterpene at SMEAR II. Although we identified several CHON^+^ fragments during the ACMCC pON experiment, and some of them as well at SMEAR II, none of them are good candidates for marker fragments for specific pON in this study. Indeed, the resolution of the LToF-AMS is high enough to unambiguously identify small fragments with high precision, but nevertheless, this information alone did not increase our knowledge of pON since all of the observed CHON^+^ fragments behaved in an identical manner, closely tracking the variations of total organics.

Furthermore, we found that using W^+^ ions in the peak width (PW) determination can greatly affect the identification of pON fragments. Although this affects all ions, we put emphasis on the organic nitrogen-containing fragments (i.e., CHON^+^ fragments). If using W^+^ ions for the PW determination during the AMS HR analysis, the PW function gets narrower than it should be. Therefore, using W^+^ ions, one is more likely to fit more (CHON^+^) fragments to improve the residual during the HR analysis. As this effect might be instrument-specific and tuning-dependent, we encourage all AMS users to investigate how much the usage of W^+^ ions affects the PW and therefore the ion identification for their own AMS instrument. As direct pON measurements are not possible with the AMS, but its data is used for calculating pON, it is of great importance to reduce uncertainties at all stages. Even though we do not have a detailed understanding of all pON formation mechanisms, this study shows that pON is an important SOA constituent and serves as a direct link between anthropogenic and biogenic emissions. While not considered useful in this study at a boreal forest site, the CHON^+^ analysis from the LTOF-AMS may prove more useful in environments with more variability in OA source types, where they might be used as markers for, e.g., pON from biomass burning. Future studies in different locations will clarify the final utility of this type of analysis.

## Data Availability

Data are available upon request by contacting the corresponding author.

## Supporting Information Available

Experimental setup used during the ACMCC pON experiment (Figure S1); time series of PW for all ions used for the PW determination for the SMEAR II data (Figure S2); difference between mass spectra of data from the ACMCC pON experiment with different data analysis scenarios (with or without W^+^ ions during the PW determination) (Figure S3); contribution of each organic family to the total organic signal for each pON precursor during the ACMCC pON experiment, with and without W^+^ ions during the PW determination (Figure S4); mass spectra of guaiacol/limonene/β-pinene + NO_3_ radicals from the ACMCC pON experiment and SMEAR II (Figure S5); examples of fitted CHON^+^ fragments from the ACMCC data (Figure S6); time series of frac_pON,NO_3__, calculated by the NO^+^/NO_2_^+^ ratio and the UMR proxy (ratio of *m*/*z* 30 and *m*/*z* 46) and the ratios of CH_2_O^+^/NO^+^ and of CH_2_O_2_^+^/NO_2_^+^ (Figure S7); two largest CHON^+^ fragments detected at SMEAR II: CH_3_NO^+^ and C_5_H_3_NO_4_^+^ (Figure S8); scatter plot of Org vs pON at SMEAR II (Figure S9); diurnal trends of pON_org_/Org and pON/total mass at SMEAR II (Figure S10); diurnal trend of the temperature (Figure S11); diurnal trend of NO*_x_*, NO, and O_3_ (Figure S12); and list of all detected CHON^+^ fragments from the SMEAR II campaign and ACMCC pON experiment (Table S1) (PDF)

## Notes

A.L. and L.R.W. are employees at Aerodyne Research, Inc., which manufactures the LToF-AMS and the PAM-OFR.
