# Supplementary material for: Detecting and Characterizing Particulate Organic Nitrates with an Aerodyne Long-ToF Aerosol Mass Spectrometer
Source: ACS Earth Space Chem. 2022 Dec 22;7(1):230–42. doi: 10.1021/acsearthspacechem.2c00314 (PMC9869397; doi:10.1021/acsearthspacechem.2c00314)
Supplement: Supplementary file 1 — sp2c00314_si_001.pdf [file sp2c00314_si_001.pdf]

Supporting Information

Detecting and Characterizing Particulate Organic Nitrates with an Aerodyne Long-ToF Aerosol Mass Spectrometer

Frans Graeffe[1], Liine Heikkinen[1,2], Olga Garmash[1,3#], Mikko Äijälä[1†], James Allan[4], Anaïs Feron[5], Manuela Cirtog[5], Jean-Eudes Petit[6], Nicolas Bonnaire[6], Andrew Lambe[7], Olivier Favez[8], Alexandre Albinet[8], Leah R. Williams[7], Mikael Ehn[1*]*

[1]Institute for Atmospheric and Earth System Research/Physics, Faculty of Science, University of Helsinki, Helsinki, 00014, Finland

[2]Department of Environmental Science and Bolin Centre for Climate Research, Stockholm University, Stockholm SE-10691, Sweden

[3]Aerosol Physics Laboratory, Physics Unit, Tampere University, Tampere, 33014, Finland

[4]Department of Earth and Environmental Sciences and National Centre for Atmospheric Science (NCAS), University of Manchester, Oxford Road, Manchester M13 9PL, UK

[5] Univ Paris Est Créteil and Université Paris 807 Cité, CNRS, LISA, Paris F-94010, France

[6]Laboratoire des Sciences du Climat et de l'Environnement (LSCE), Gif-sur-Yvette 91191, France

[7]Aerodyne Research Inc., Billerica, Massachusetts 01821, USA

[8]Institut National de l'Environnement Industriel et des Risques (INERIS), Verneuil-en-Halatte 60550, France

Present Addresses:

[#]Department of Atmospheric Sciences, University of Washington, Seattle, Washington 98195, United States

[†]School of Energy Systems (LES), Lappeenranta-Lahti University of Technology (LUT), Lappeenranta 53850, Finland

*Corresponding authors: Frans Graeffe (frans.graeffe@helsinki.fi) and Mikael Ehn (mikael.ehn@helsinki.fi)

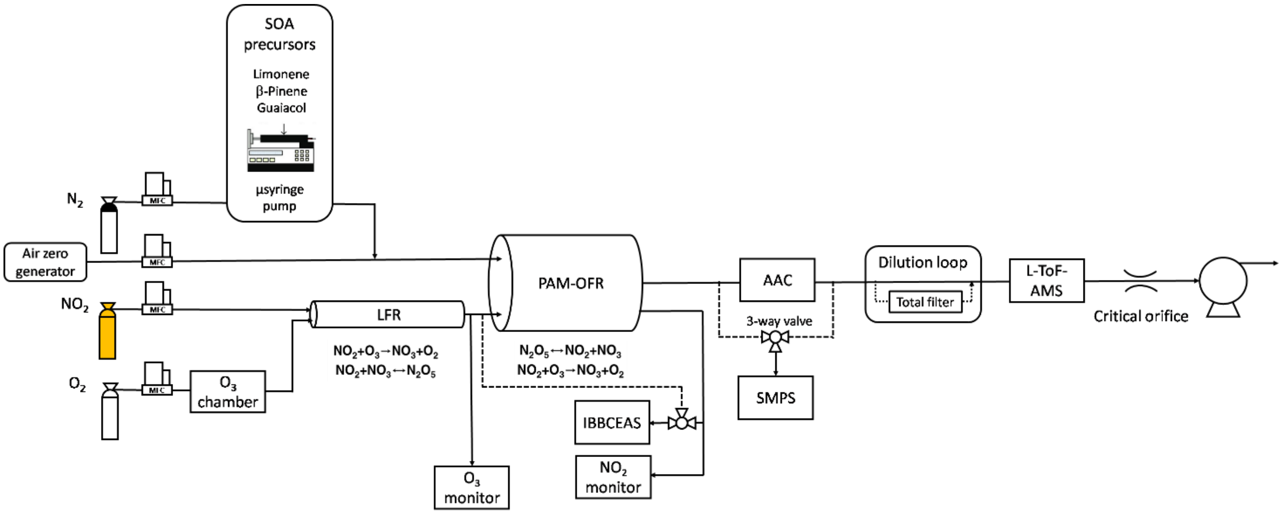

Figure S1. Simplified representation of the experimental setup used during the ACMCC pON experiment. Constant SOA generation was obtained using a PAM-OFR. The overall setup includes a constant SOA precursor introduction (liquid compounds) and continuous generation of N_2O_5 and NO_3 radicals through a LFR (OFR-iN_2O_5 method). An IBBCEAS was used to monitor NO_3 radical concentrations generated. An AAC selected monodisperse aerosol, monitored using a SMPS, before being measured by the LToF-AMS. The "dilution loop" provided different stable SOA concentration levels analysed by the LToF-AMS.

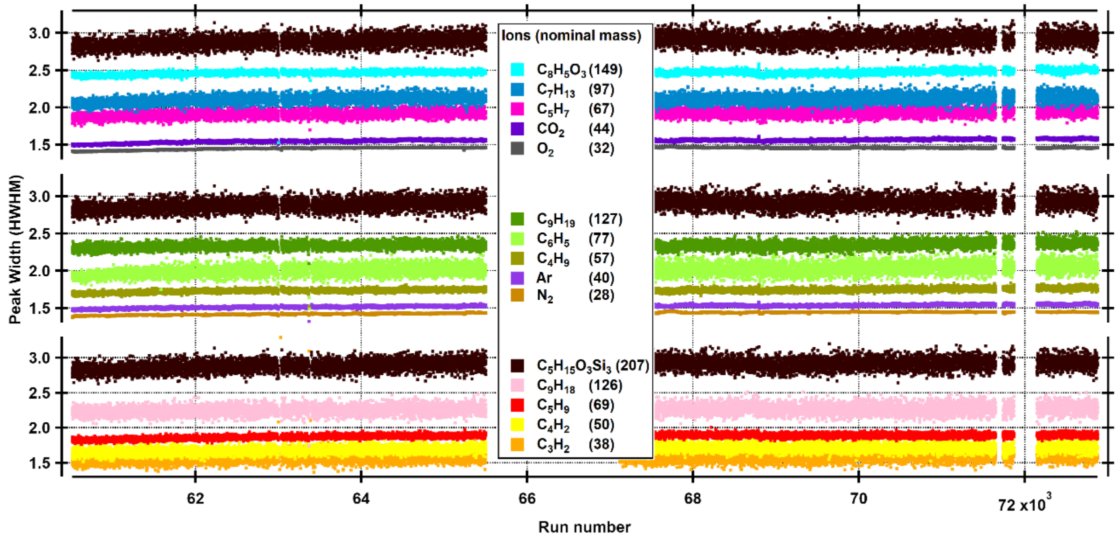

Figure S2. Time series of peak widths (PW) for all ions used for the PW determination for the SMEAR II data.

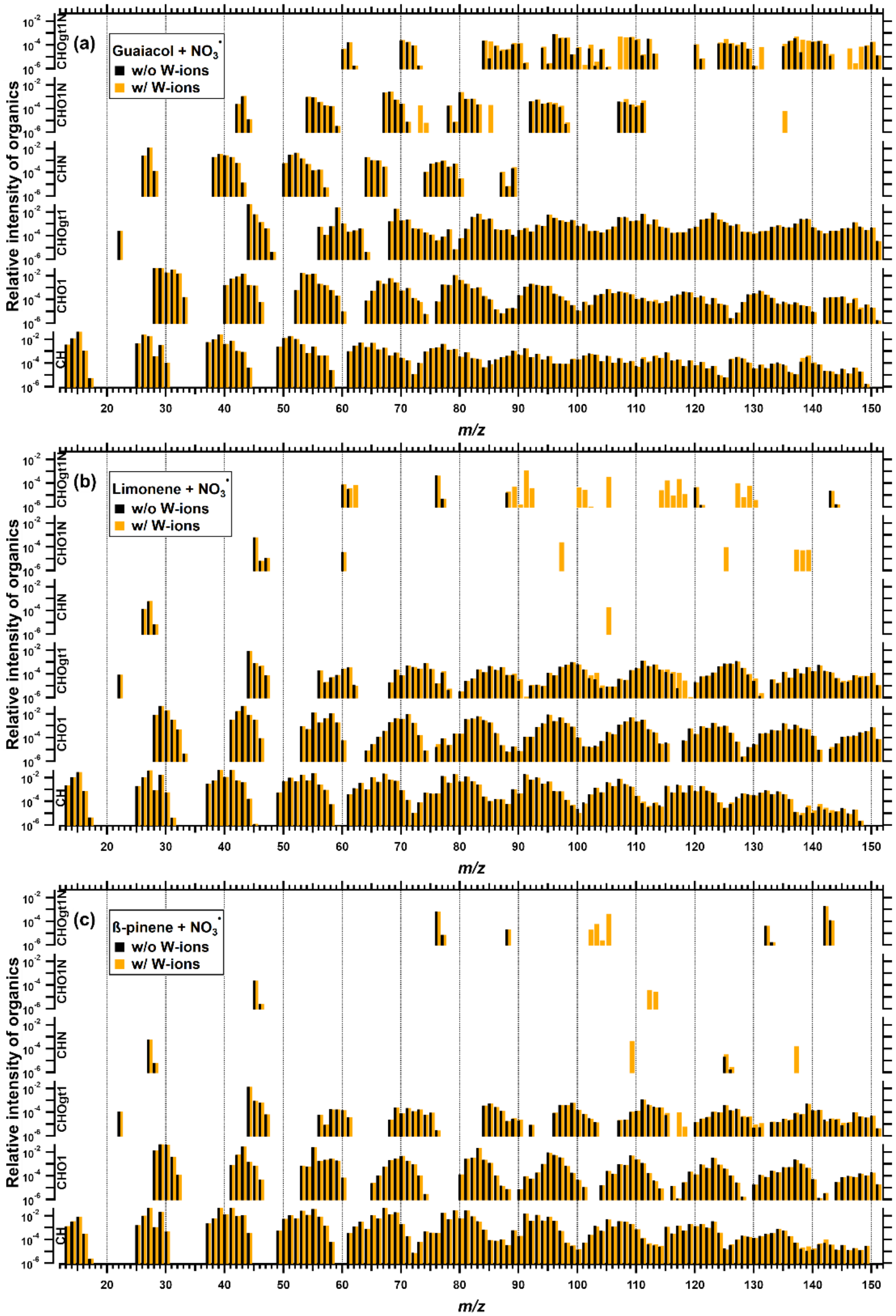

Figure S3. The difference between mass spectra of **(a)** guaiacol, **(b)** limonene **(c)** β-pinene + NO_3 radicals from the ACMCC pON experiment with different data analysis scenarios. Data where W^+ ions are not used (w/o W^+ ions) for the PW determination are black and where W^+ ions are used

(w/ W^+ ions) are in orange. Each organic family is displayed on a separate y-axis. The *m/z*-axis for w/ W^+ ions data has an offset of 0.3 for visualisation reasons.

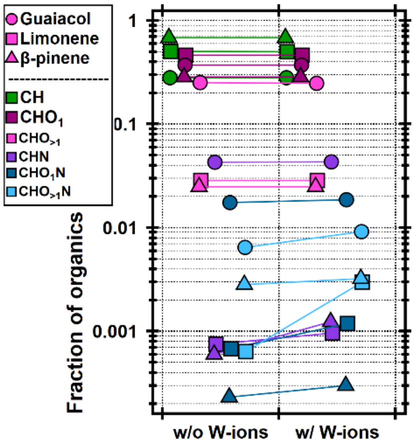

Figure S4. The contribution of each organic family to the total organic signal for each pON precursor during the ACMCC pON experiment for the two cases; when W^+ ions are not used and are used during the PW determination.

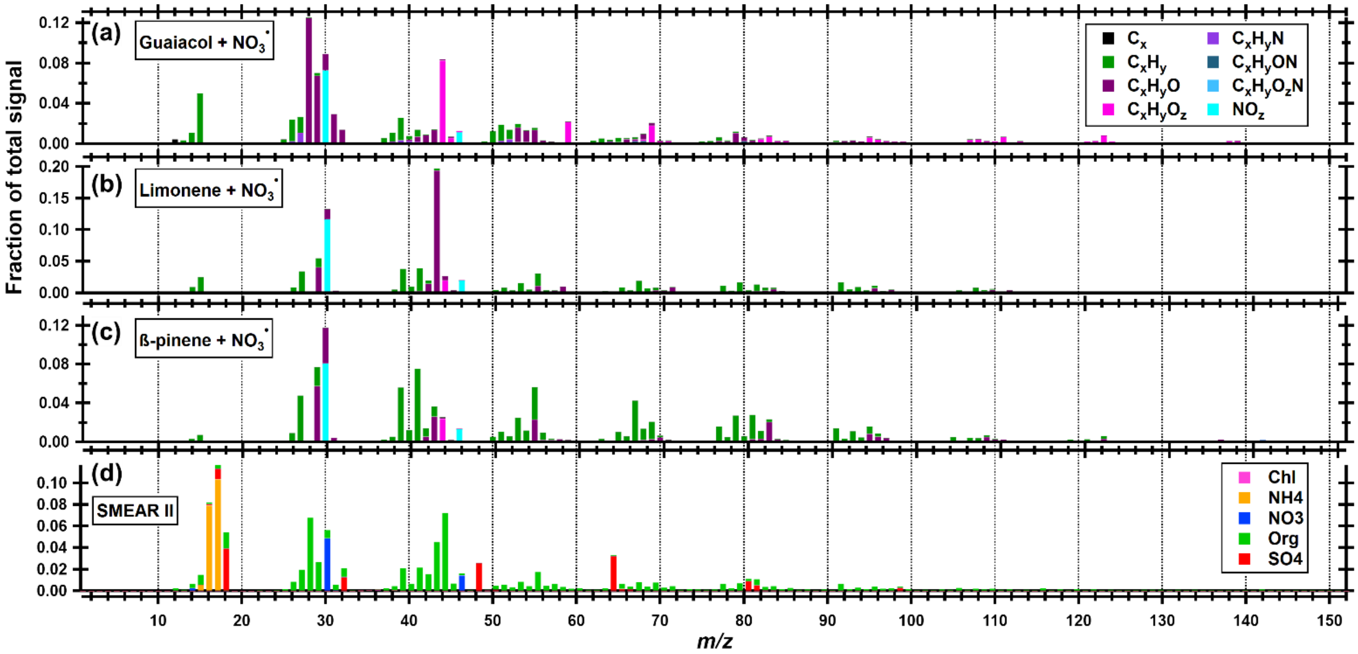

Figure S5. Mass spectra of **(a)** guaiacol, **(b)** limonene, **(c)** β-pinene + NO_3 radicals from the ACMCC pON experiment and (d) SMEAR II. Note: different signals on same *m/z* value are stacked on each other.

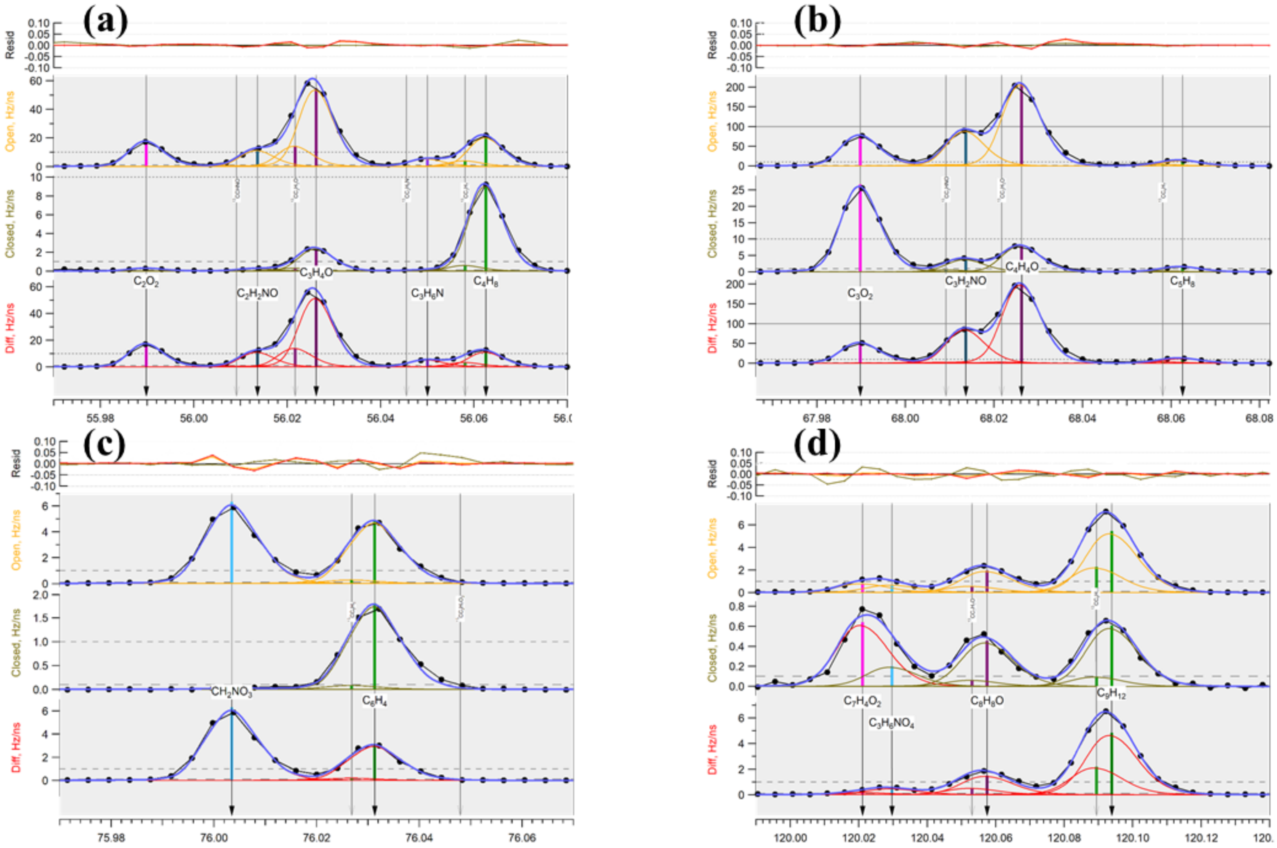

Figure S6. Examples of fitted CHON$^+$ fragments from the ACMCC data: $C_2H_2NO^+$ (at *m/z* 56) **(a)** and $C_3H_2NO^+$ (at *m/z* 68) **(b)** are from the guaiacol SOA, $CH_2NO_3^+$ (at *m/z* 76) **(c)** is from the β-pinene SOA and $C_3H_6NO_4^+$ (at *m/z* 120) **(d)** is from the limonene SOA.

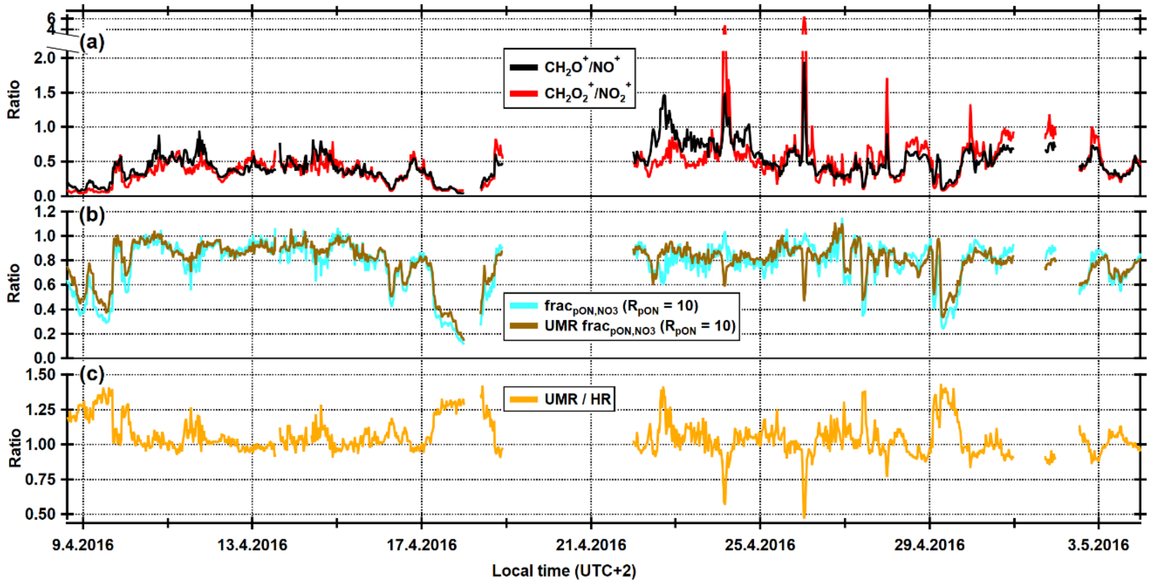

Figure S7. Time series of **(a)** the ratios of CH_2O^+/NO^+ (at *m/z* 30) and of $CH_2O_2^+/NO_2^+$ (at *m/z* 46), **(b)** $frac_{pON,\ NO_3}$, calculated by the NO^+/NO_2^+ ratio and the UMR proxy (ratio of *m/z* 30 and *m/z* 46) and **(c)** the ratio of the UMR and HR methods. .

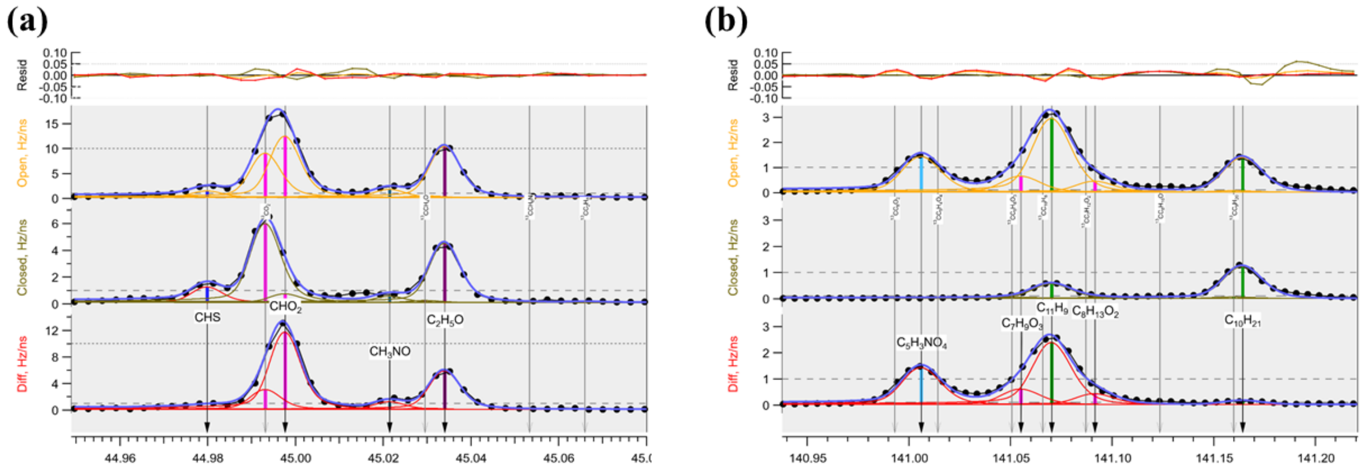

Figure S8. The two largest $CHON^+$ fragments detected at SMEAR II: CH_3NO^+ (at *m/z* 45) **(a)** and $C_5H_3NO_4^+$ (at *m/z* 141) **(b)**.

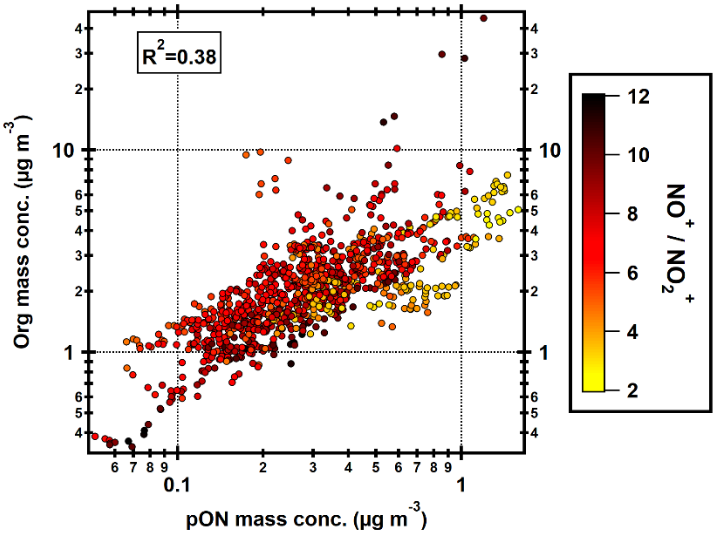

Figure S9. Scatter plot of Org vs pON at SMEAR II. The colour scale show the NO^+/NO_2^+ ratios in which yellow colours indicate the presence of inorganic ammonium nitrate. The Pearson correlation coefficients (squared) are shown in each subplot. Note that the data are displayed in log-log scales.

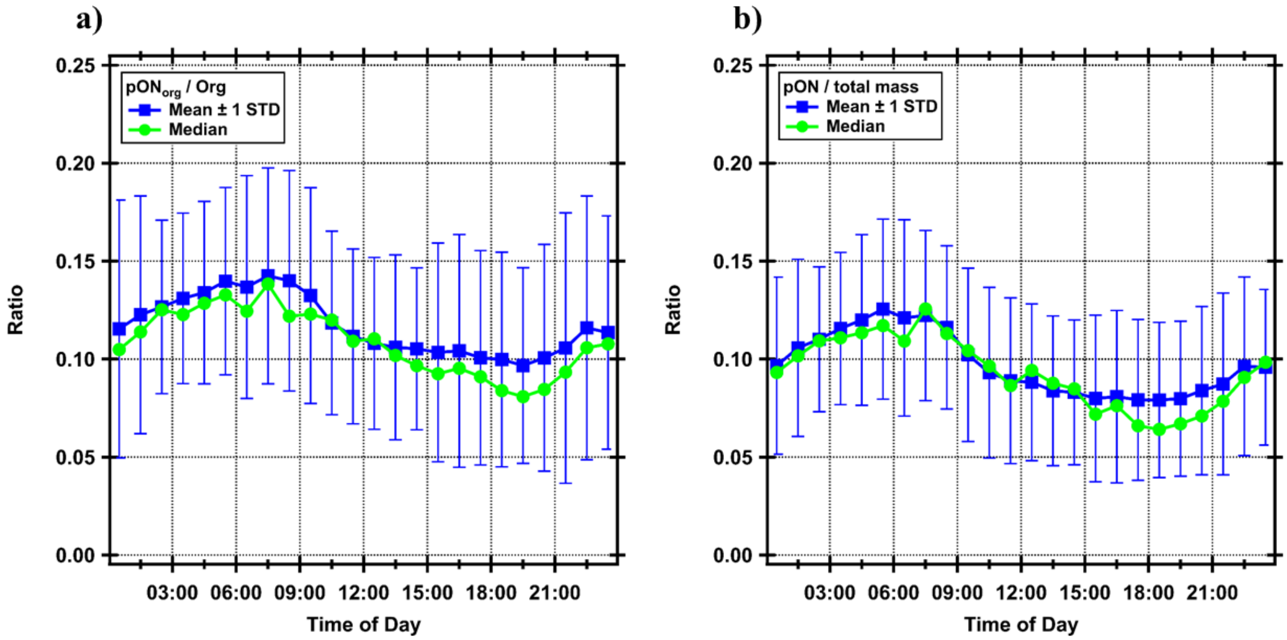

Figure S10. Diurnal trends of pON_{org} / organic fraction **(a)** and pON / total mass fraction **(b)** at SMEAR II.

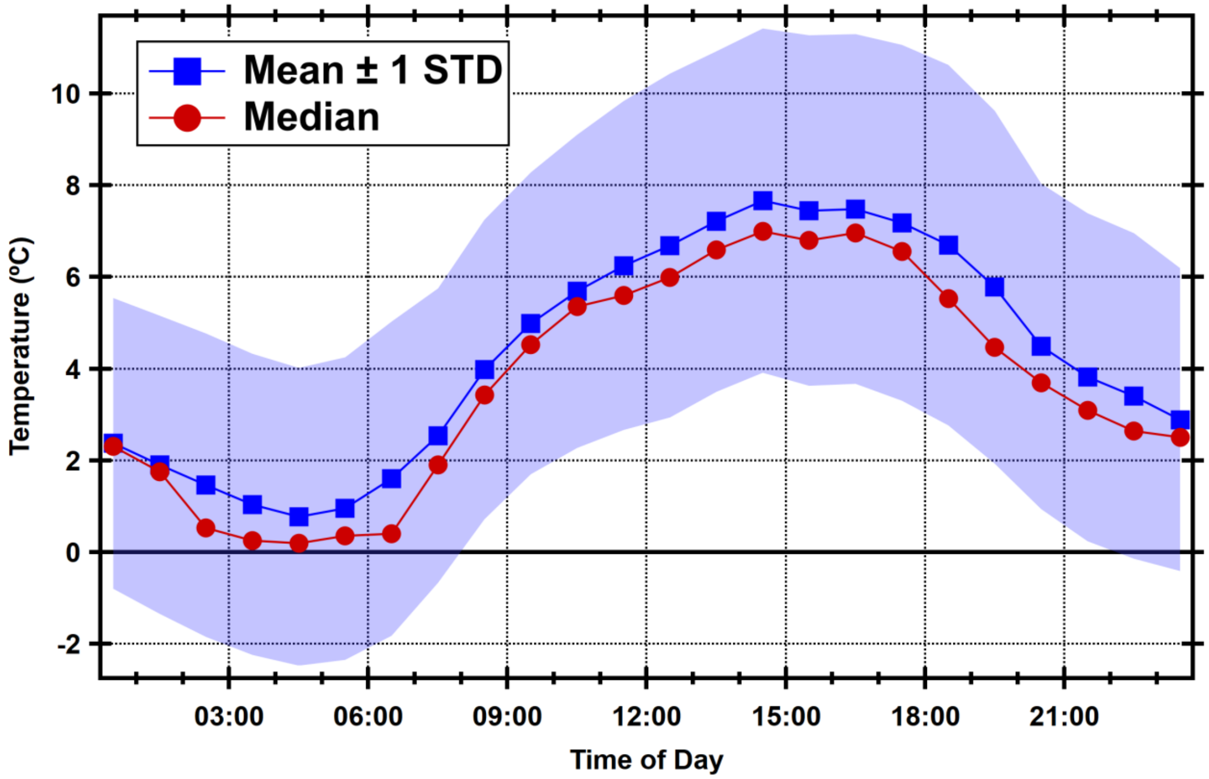

Figure S11. Diurnal trend of the temperature at SMEAR II.

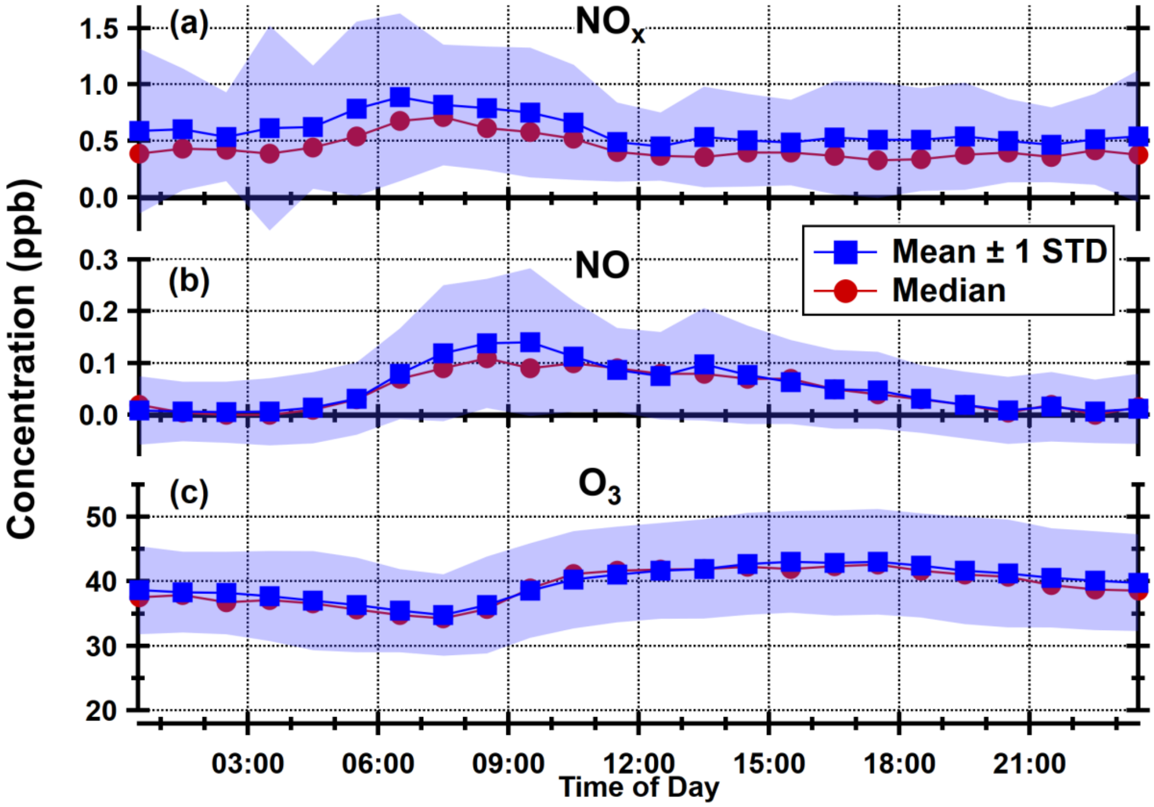

Figure S12. Diurnal trends of **(a)** NO_x, **(b)** NO and **(c)** O_3 at SMEAR II.

Table S1. List of all detected $CHON^+$ fragments from the SMEAR II campaign and ACMCC pON experiment. All ions are positively charged.

m/z	Guaiacol	Limonene	β-pinene	SMEAR II
42	CNO			
43	CHNO			
45		CH_3NO	CH_3NO	CH_3NO
47		CH_5NO		
54	C_2NO			

55	C_2HNO			
56	C_2H_2NO			
57	C_2H_3NO			
58	C_2H_4NO			
59				C_2H_5NO
60	CH_2NO_2	C_2H_6NO<br>CH_2NO_2		C_2H_6NO
61	CH_3NO_2	CH_3NO_2		
67	C_3HNO			
68	C_3H_2NO			
69	C_3H_3NO			
70	C_3H_4NO<br>C_2NO_2			
71	C_2HNO_2			
72	$C_2H_2NO_2$			C_3H_6NO
74				$C_2H_4NO_2$
75				$C_2H_5NO_2$
76		CH_2NO_3	CH_2NO_3	
78	C_4NO			
80	C_4H_2NO			
81	C_4H_3NO			
82	C_4H_4NO			
83	C_4H_5NO			
84	$C_3H_2NO_2$			
86	$C_3H_4NO_2$			
87	$C_3H_5NO_2$			

88	$C_3H_6NO_2$	$C_3H_6NO_2$	$C_3H_6NO_2$	$C_3H_6NO_2$
89	$C_2H_3NO_3$<br>$C_3H_7NO_2$			
90	$C_2H_4NO_3$<br>$C_3H_8NO_2$			
92	C_5H_2NO			
93	C_5H_3NO			
94	C_5H_4NO<br>C_4NO_2			
95	C_5H_5NO			
96	$C_4H_4N_2O$<br>$C_4H_2NO_2$			
97	$C_4H_5N_2O$<br>$C_4H_3NO_2$			
98	$C_4H_4NO_2$			
100	$C_3H_2NO_3$			$C_5H_{10}NO$<br>$C_4H_6NO_2$
102	$C_3H_4NO_3$			
104	$C_3H_6NO_3$			
107	C_6H_5NO			
108	C_6H_6NO			
109	C_6H_7NO<br>$C_5H_3NO_2$			
110	$C_5H_6N_2O$<br>$C_5H_4NO_2$			
111	$C_5H_7N_2O$			

112	$C_5H_6NO_2$			
114				$C_5H_8NO_2$
120	$C_6H_2NO_2$	$C_3H_6NO_4$		
124	$C_5H_2NO_3$			
125	$C_5H_3NO_3$			
126	$C_5H_4NO_3$			
127	$C_5H_5NO_3$			
128	$C_4H_2NO_4$<br>$C_5H_6NO_3$			
129	$C_4H_3NO_4$			
132			$C_4H_6NO_4$	
133				$C_4H_7NO_4$
134				$C_4H_8NO_4$
135	$C_7H_5NO_2$			
136	$C_6H_2NO_3$<br>$C_7H_6NO_2$			
137	$C_6H_3NO_3$<br>$C_7H_7NO_2$			
139				$C_7H_9NO_2$
140	$C_5H_2NO_4$<br>$C_6H_6NO_3$			
141	$C_5H_3NO_4$<br>$C_5H_5N_2O_3$<br>$C_6H_7NO_3$			$C_5H_3NO_4$
142	$C_5H_4NO_4$		$C_6H_8NO_3$	$C_5H_4NO_4$<br>$C_6H_8NO_3$

143	$C_5H_5NO_4$	$C_7H_{13}NO_2$		
146				$C_5H_8NO_4$
147				$C_5H_9NO_4$
149	$C_7H_3NO_3$			
150	$C_7H_4NO_3$			
